# Polyphenol‐Mediated Multifunctional Human–Machine Interface Hydrogel Electrodes in Bioelectronics

**DOI:** 10.1002/smsc.202400362

**Published:** 2024-11-21

**Authors:** Lili Jiang, Donglin Gan, Chuangyi Xu, Tingting Zhang, Mingyuan Gao, Chaoming Xie, Denghui Zhang, Xiong Lu

**Affiliations:** ^1^ Institute of Medical Industrial and Information Technology College of Information Science and Technology Zhejiang Shuren University Hangzhou Zhejiang 310015 China; ^2^ Institute of Biomedical Engineering College of Medicine Southwest Jiaotong University Chengdu Sichuan 610031 China; ^3^ Jiangsu Collaborative Innovation Center of Biomedical Functional Materials Jiangsu Key Laboratory of Bio‐functional Materials School of Chemistry and Materials Science Nanjing Normal University Nanjing 210023 China; ^4^ School of Traffic & Transportation Engineering Central South University Changsha 410000 China; ^5^ College of Engineering and Technology Southwest University Chongqing 400716 China; ^6^ YiBin Research Institute Southwest Jiaotong University Chengdu Sichuan 610031 China

**Keywords:** human–machine interfaces, hydrogels, multifunctions, polyphenol‐mediated bioadhesions

## Abstract

Human–machine interface (HMI) electrodes enable interactions between humans and bioelectronic devices by facilitating electrical stimulation and recording neural activity. However, reconciling the soft, hydrated nature of living human tissues with the rigid, dry properties of synthetic electronic systems is inherently challenging. Overcoming these significant differences, which is critical for developing compatible, effective, and stable interfaces, has become a key research area in materials science and technology. Recently, hydrogels have gained prominence for use in HMI electrodes because these soft, hydrated materials are similar in nature to human tissues and can be tuned through the incorporation of nanofillers. This review examines the functional requirements of HMI electrodes and highlights recent progress in the development of polyphenol‐mediated multifunctional hydrogel‐based HMI electrodes for bioelectronics. Furthermore, aspects such as mussel‐inspired and polyphenol‐mediated adhesion, underlying mechanisms, tissue‐matching mechanical properties, electrochemical performance, biocompatibility, biofouling resistance, stability under physiological conditions, anti‐inflammatory, and antioxidant properties are discussed. Finally, applications in bioelectronics and further perspectives are outlined. Advances in HMI hydrogel electrodes are expected to facilitate the unprecedented integration of biological systems and electronic devices, potentially revolutionizing various biomedical fields and enhancing the capabilities and performance of bioelectronic devices.

## Graphical abstract

This review focuses on the development of polyphenol‐mediated multifunctional hydrogel‐based human–machine interface electrodes for bioelectronics. Key functions discussed include mussel‐inspired and polyphenol‐mediated adhesion, underlying mechanisms, tissue‐matching mechanical properties, electrochemical performance, biocompatibility, biofouling resistance, stability under physiological conditions, as well as anti‐inflammatory and antioxidant properties. Applications in bioelectronics are explored, and future perspectives are outlined.

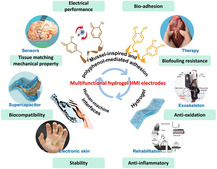

## 1 Introduction

Human–machine interfaces (HMIs) are systems that allow humans to interact with machines, particularly computers or other electronic devices, through a combination of hardware and software.^[^ ^1^ ^]^ The first HMIs were early communication devices such as the telegraph and telephone, and subsequent progress has led from simple control systems to sophisticated, intuitive interfaces that facilitate seamless interactions between human physiology and machines.^[^ ^2^ ^]^ Since the discovery of bioelectricity in Luigi Galvani's landmark experiment, a better understanding of electronic communication between human biology and electronics, so‐called bioelectronics, has been an ambitious and ongoing application of HMI technology.^[^ ^3^ ^]^ In bioelectronics, HMIs play a crucial role in the development of devices that can accurately sense, process, and respond to biological signals, thereby enabling seamless communication between the human body and electronic devices.

HMI electrodes in direct contact with biological tissues that detect and transmit bioelectrical signals from the body to a machine, or vice versa, are the most critical components in bioelectronics ranging from medical devices to wearable technology and brain–computer interfaces (BCIs).^[^ ^4^ ^]^ HMI electrodes have been carefully chosen to collect and deliver various bioelectronic signals to different parts of the human body, such as the skin, brain, spinal cord, and heart. In particular, various HMI electrodes incorporated into commercial wearable epidermal bioelectronic devices have been routinely used for diverse clinical purposes, including deep brain stimulation probes for Parkinson's disease and essential tremors, neural interfaces for robotic prostheses, flexible electrode arrays for heart failure, and closed‐loop electrode arrays for spinal cord injuries.^[^ ^5^ ^]^

Despite remarkable advances over the past few decades, the intrinsic differences between human tissues and manmade electronics complicate the design and manufacture of HMI electrode materials for next‐generation bioelectronics. First, the human body and tissues, including the skin, muscles, heart, spinal cord, and brain, are typically soft, with tissues having Young's moduli of 0.005–0.1 MPa.^[^ ^6^ ^]^ In contrast, HMI electrodes, which commonly consist of metals (silver/silver chloride, gold, platinum, stainless, and titanium) or silicon, are rigid and static, with Young's moduli of 70–210 GPa.^[^ ^7^ ^]^ Second, tissues are highly hydrated, containing both extracellular and intracellular fluids.^[^ ^8^ ^]^ Moreover, these extracellular fluids and intracellular environments are rich in ions such as sodium (Na^+^), potassium (K^+^), calcium (Ca^2+^), and chloride (Cl^−^), which is a fundamental requirement for the generation and transfer of bioelectronic signals.^[^ ^9^ ^]^ In contrast, in conventional dry HMI electrodes, electrons serve as carriers for collecting and transporting electronic signals. These mismatches highlight the inherent difficulties in signal transfer between human tissues and electronic systems faced by HMI electrodes.

In addition, HMI electrodes can suffer from mechanical and signal transmission differences between human tissues and rigid materials. For instance, during normal postural movements, the skin, muscles, spinal cord, and peripheral nerves can endure up to 30% tensile strain and displacement.^[^ ^10^ ^]^ During cardiovascular activity, the heart and vascular tissues undergo continuous periodic mechanical deformation.^[^ ^11^ ^]^ The resulting mismatch between rigid materials and human tissues leads to poor mechanical interactions and adverse physiological reactions, including skin irritation, allergic reactions, and inflammation, thereby limiting seamless communication and the reliability of HMIs. Moreover, gravity contributes to the mechanical environment experienced by tissues. HMI electrodes in direct contact with these tissues are influenced by the same mechanical environment. Consequently, static HMI electrodes may slip or move out of place, particularly if worn on body parts that are prone to movement or perspiration. Such movement can affect electrode stability and performance, leading to inconsistent data collection or signal loss. Thus, secure bioadhesion of HMI electrodes to tissues in the water‐rich environment of the human body is essential to ensure reliable performance and enhance user comfort.

Hydrogels, which are composed of crosslinked polymer networks infiltrated with water, have been extensively investigated in tissue engineering and biomedicine due to their similarities to biological tissues.^[^ ^12^ ^]^ The soft and flexible nature of hydrogels minimizes mechanical mismatch with biological tissues, whereas their high water content provides a wet, ion‐rich physiological environment. Moreover, the electrical, mechanical, bioadhesive, and biological properties of hydrogels can be tuned, providing versatile bridging materials that can be integrated into biological systems.^[^ ^13^ ^]^ Because of these advantages, hydrogels have recently garnered attention in the field of bioelectronics, contributing to ongoing efforts to establish seamless interfaces between biological and electronic systems.

With the development of bioelectronic devices, hydrogel‐based HMI electrodes have been integrated to enhance their interfacing with the human body. Natural polymer‐based hydrogel electrodes (such as alginate,^[^ ^14^ ^]^ chitosan,^[^ ^15^ ^]^ cellulose,^[^ ^16^ ^]^ and gelatin^[^ ^17^ ^]^) offer high biocompatibility but limited tunability, which restricts their application in HMI electrodes.^[^ ^18^ ^]^ In contrast, synthetic polymer‐based hydrogels, such as polyvinyl alcohol (PVA)^[^ ^19^ ^]^ and polyacrylamide (PAM),^[^ ^20^ ^]^ can be easily tailored, making their tunable chemical and physical properties suitable for multifunctional hydrogel‐based HMI electrodes. However, concerns regarding its biocompatibility persist.^[^ ^21^ ^]^ Consequently, double networks and composite hydrogels have emerged as effective solutions for balancing various performance attributes.^[7a]^ Despite these advances, incorporating these electrodes into existing devices to enhance the patient experience while maintaining device performance remains challenging. Further innovations in the design and fabrication of hydrogel‐based HMI electrodes are essential for creating multifunctional and sophisticated interfaces between the human body and future bioelectronic systems.

**Figure 1:**
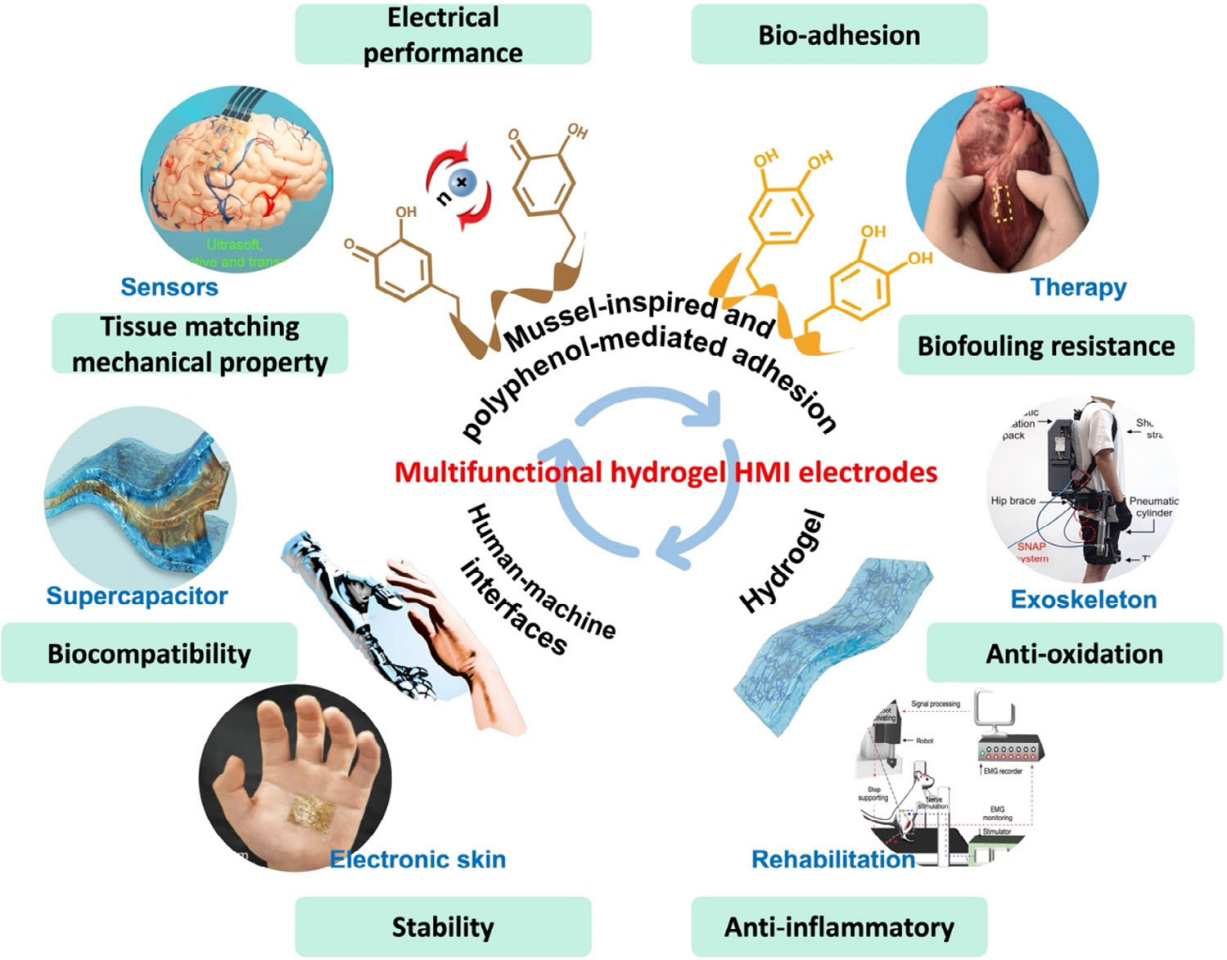
Graphical overview of this review. Section 2 introduces mussel‐inspired and polyphenol‐mediated adhesion. Section 2.1, 2.2, 2.3, 2.3.1, 2.3.2, 2.3.3, 2.3.4 summarize the required functions of hydrogel‐based HMI electrodes for bioelectronics. Section 10 presents recent applications of polyphenol‐mediated and mussel‐inspired hydrogel‐based HMI electrodes for bioelectronics. Section 11 provides remaining challenges and perspectives. The therapy, electronic skin, triboelectric nanogenerator (TENG), exoskeleton, and closed‐loop robot‐assisted rehabilitation photographs are reproduced with permission,^[^ ^152^, ^153^, ^154^, ^155^, ^156^ ^]^ respectively. The therapy photograph is reproduced with permission.^[^ ^152^ ^]^ Copyright 2022, Wiley. The electronic skin photograph is reproduced with the permission.^[^ ^153^ ^]^ Copyright 2023, Wiley. The TENG photograph is reproduced with the permission.^[^ ^154^ ^]^ Copyright 2023, ACS Publications. The exoskeleton photograph is reproduced with the permission.^[^ ^155^ ^]^ Copyright 2024, Science. The rehabilitation photograph is reproduced with the permission.^[^ ^156^ ^]^ Copyright 2023, Nature.

This review aims to provide a comprehensive set of rational design guidelines for advanced HMI electrodes in bioelectronic applications, with a focus on the multifunctional performance requirements of both users and devices. First, mussel‐inspired and polyphenol‐mediated adhesion, a recent focus in hydrogel‐based HMI electrode research, is discussed. Subsequently, recent advances in HMI hydrogel electrodes are categorized into eight classes based on function: 1) polyphenol‐mediated adhesion; 2) mechanical properties; 3) electrical performance; 4) biocompatibility; 5) biofouling resistance; 6) stability under physiological conditions; 7) anti‐inflammatory properties; and 8) antioxidant properties. Furthermore, the advanced applications of hydrogel‐based HMI electrodes in bioelectronics are explored (**Figure** **1**). Finally, several directions and guidelines for the future development of HMI hydrogel electrodes are proposed before remaining opportunities and challenges are summarized. The multifunctional design of hydrogel‐based HMI electrodes is emphasized, with the goal of continually improving the capabilities and user comfort of these electrodes, thereby expanding their impact and accessibility.

## 2 Mussel‐Inspired Adhesion

### 2.1 Mussel Adhesion Chemistry

To bridge the gap between electronic devices and biological systems, effective adhesion of HMI electrodes is vital for the performance and reliability of bioelectronic devices. Drawing inspiration from nature, spider webs can cling to walls, but this strong adhesion is lost upon dissolution in water. In contrast, mussels are capable of strong underwater adhesion, which is unaffected by heavy waves or storms. Mussels adhere to various surfaces, such as rocks and ship hulls, by secreting mussel foot proteins (Mfps), including Mfp‐2, Mfp‐3, Mfp‐4, Mfp‐5, and Mfp‐6. Mfps form tough fibers called byssus that can attach to different surfaces.^[^ ^22^ ^]^ These proteins, particularly Mfp‐3 and Mfp‐5, contain the amino acids 3,4‐dihydroxyphenylalanine (Dopa) and lysine, which have been demonstrated to provide strong adhesion.

**Figure 2:**
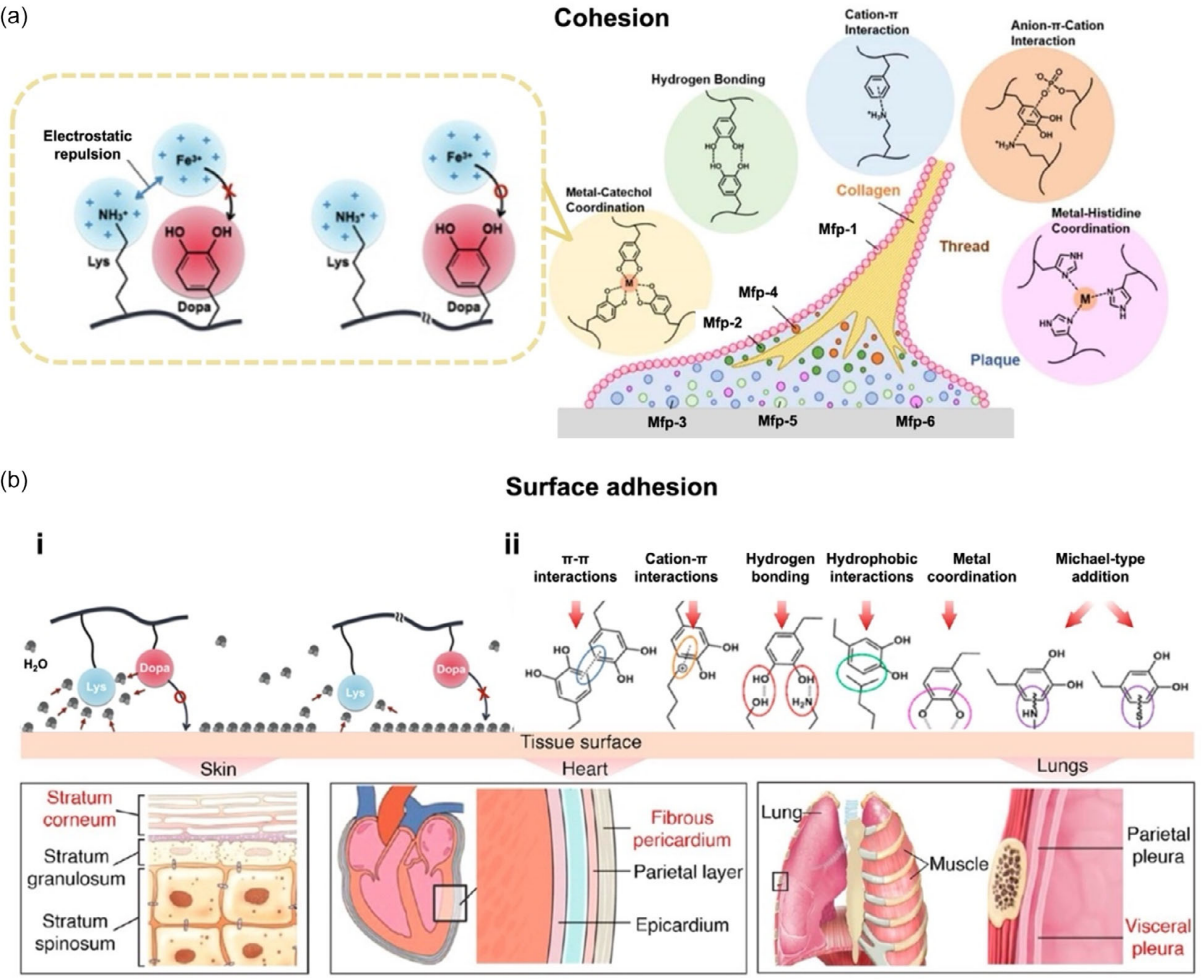
Mussel adhesion mechanisms. a) Cohesion and adhesion of mussels. Reproduced with permission.^[^ ^24^ ^]^ Copyright 2023, Frontiers. The enlarged region shows the effects of lysine on cohesion. Reproduced with permission.^[^ ^26^ ^]^ Copyright 2020, Elsevier. b) Mussel‐inspired surface adhesion mechanisms: i) adhesion in a wet environment. Reproduced with permission.^[^ ^26^ ^]^ Copyright 2022, Elsevier; ii) adhesion on tissue surfaces. Reproduced with permission.^[^ ^29a^ ^]^ Copyright 2021, Elsevier and ^[^ ^29b^ ^]^ Copyright 2013, OpenStax.

Dopa has numerous catechol groups, which can act as adhesives by forming reversible, noncovalent bonds through hydrogen bonding, π–π stacking, cation–π interactions, or coordination with metal oxides. These interactions contribute to both byssus cohesion and adhesion to surfaces.^[^ ^23^ ^]^ Catechol groups can also chelate metal ions such as Fe^3+^, form dynamic borate bonds with boric acid groups, and undergo disproportionation reactions to enhance the adhesion strength (**Figure** **2**a).^[^ ^24^ ^]^ As the cohesion of Dopa alone cannot maintain long‐term mussel adhesion, the processes involved in overcoming the adverse effects of Dopa oxidation have attracted much interest. In their natural environment, mussels cosecrete H_3_O^+^ and Mfps into the distal grooves. Consequently, despite being surrounded by seawater, a lower pH is maintained within the adhesive plaques, which inhibits Dopa oxidation. Additionally, antioxidant proteins within mussels, such as Mfp‐6, contain cysteine thiols that can reduce dopaquinone to Dopa. This dynamic redox behavior effectively prevents oxidation, ensuring the dynamic and sustained adhesion of mussels to various surfaces.^[^ ^25^ ^]^

Lysine, which carries a positive charge, can act as an auxiliary by displacing surface salt ions to enhance adhesion. Adhesion can also be strengthened through electrostatic interactions. In certain scenarios, cations can form ionic bonds with negatively charged surfaces, and these electrostatic interactions are critical in high‐ionic‐strength environments such as seawater. In addition, as lysine attracts water molecules, interactions with hydrated surface cations could facilitate their removal. This process allows catechol groups to approach substrate surfaces more closely.^[^ ^24^, ^26^ ^]^ Such proximity enables stronger interactions between catechol groups and surfaces through hydrogen or coordination bonds, thereby significantly enhancing the adhesive properties, even in a water environment (Figure 2bi). Studies have demonstrated that lysine can only attract water molecules around Dopa to enhance underwater surface adhesion when Dopa and lysine are adjacent on Mfp‐3.^[^ ^27^ ^]^ However, this adjacent position disrupts the approach of ferric ions (Fe^2+^) both electrically and structurally, thereby hindering the curing of Dopa to some extent^[^ ^26^, ^28^ ^]^ (enlarged region, Figure 2a). The underwater adhesion mechanisms of mussels have also been extensively studied for applications in human tissue adhesion. For this purpose, the catechol functional groups of Dopa can also contribute to tissue adhesion through noncovalent bonding (Figure 2bii).^[^ ^29^ ^]^

### 2.2 Polyphenol‐Mediated Adhesion

As demonstrated in mussels, Dopa, as a polyphenol, plays a crucial role in natural adhesion mechanisms. Hence, polyphenol‐mediated adhesion has garnered significant interest for imparting adhesive properties to materials.^[^ ^30^ ^]^ Inspired by the mussel adhesion mechanisms, polyphenol‐mediated adhesion has been increasingly used in the design of hydrogel‐based HMI electrodes for bioelectronics.

**Figure 3:**
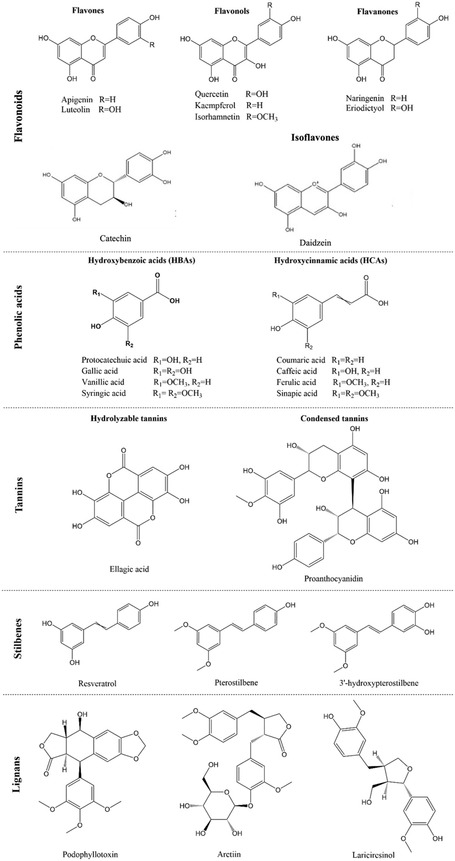
Structures of phenolic compounds. Reproduced with permission.^[^ ^32a^ ^]^ Copyright 2022, Nature and ^[^ ^32b^ ^]^ Copyright 2023, MDPI.

Phenolic compounds, a diverse class of plant secondary metabolites with high biocompatibility that are widely found in plant organs, marine algae, and mussels play crucial roles in various physiological processes.^[^ ^31^ ^]^ Compounds such as flavonoids, phenolic acids, tannins, stilbenes, and lignans contain numerous catechol functional groups, making them valuable adhesive building blocks (**Figure** **3**).^[^ ^32^ ^]^

Due to their comparable molecular structures, phenolic derivatives and Dopa share similar adhesion mechanisms. These compounds can interact with substrates through various covalent and noncovalent interactions mediated by catechol groups, as illustrated in Figure 2b. For HMI electrodes, the stratum corneum of the skin is the primary contact tissue. The stratum corneum is composed of dead cells densely packed with keratin proteins, which contain hydrophobic amino acids such as alanine and valine as well as hydrogen bond‐donating amino acids such as glutamic acid and serine.^[^ ^33^ ^]^ These surface components can form noncovalent π–π interactions and hydrogen bonds with phenolic compounds. Moreover, covalent bonds can be formed through Michael addition reactions of quinones with the thiol or amino side chains of amino acid residues such as cysteine and lysine. These amino acids are abundant on the surface of internal soft tissues, such as the fibrous pericardium layer of the heart and the visceral pleura layer of the lungs (Figure 2bii).^[^ ^34^ ^]^

These polyphenol‐mediated adhesion mechanisms provide inspiration for the design of multifunctional hydrogel‐based HMI electrodes. However, despite their structural similarities, the physical and chemical characteristics of phenolic compounds vary, which can result in significantly different biological responses. These differences may limit the potential applications of synthesized biomaterials. The most common phenol‐containing compounds that can be introduced into hydrogel‐based HMI electrodes for bioadhesion are discussed below.

### 2.3 Polyphenol‐Mediated Adhesive Strategies for HMI Electrodes

Recently, biomimetic strategies leveraging the natural adhesive and structural properties of polyphenols in biological systems have been applied to synthesize polyphenol‐mediated nanomaterials for hydrogel‐based HMI electrodes. These strategies aim to replicate the high adhesive strength, biocompatibility, and mechanical robustness of natural polyphenol‐containing materials such as mussel‐adhesive proteins. The following sections detail some of the most common phenolic compounds and composite nanomaterials used in the design of HMIs.

#### 2.3.1 Catecholamines

Catecholamines such as dopamine have received considerable attention for biomimetic adhesion strategies.^[^ ^35^ ^]^ Inspired by the adhesive proteins in mussels, polydopamine (PDA) is the most widely used polyphenol for imparting adhesion to hydrogels. PDA, which can be easily synthesized through self‐polymerization of dopamine under mild conditions, can form coatings on various materials, providing a universal adhesive layer.^[^ ^36^ ^]^ The amine and catechol functional groups in PDA allow for further functionalization, enabling the introduction of additional biomolecules or nanoparticles to tailor the hydrogel properties. Jung et al.^[^ ^37^ ^]^ prepared polyacrylamide (PAM)/PDA hydrogels embedded with extralarge‐pore mesoporous silica nanoparticles. The incorporation of PDA and mesoporous silica nanoparticles provided increased cohesive properties that enhanced the strength and adhesiveness to skin tissue. Our group has also conducted numerous studies on mussel‐inspired adhesion using PDA and composite nanoparticles, leveraging not only the catechol groups of PDA but also the mussel‐inspired redox environment to maintain these functional groups within hydrogel electrodes.^[^ ^38^ ^]^ For example, we developed conductive, redox‐active, water‐soluble nanosheets via the self‐assembly of poly(3,4‐ethylenedioxythiophene) (PEDOT) on a PDA‐reduced and sulfonated graphene oxide (PSGO) template. These PSGO‐PEDOT nanosheets, as nanofillers for hydrogel electrodes, were redox active, with the abundant catechol groups being maintained by dynamic redox reactions. The hydrogel adhered strongly to porcine skin (20 kPa) and exhibited a minimal decrease in adhesion strength after 30 stripping–adhesion cycles. Importantly, the hydrogel did not cause skin irritation or leave a residue on the skin after stripping.^[^ ^39^ ^]^ However, dopamine, a critical neurotransmitter, plays a significant role in various physiological processes in the brain, including those regulated by the hypothalamus, enabling chemical communication in the central nervous system.^[^ ^40^ ^]^ The mechanisms of action of dopamine involve complex interactions with dopamine receptors and other neurotransmitter systems.^[^ ^41^ ^]^ However, the potential influence of introducing dopamine or PDA into hydrogel‐based HMI electrodes on these physiological mechanisms remains unknown.

#### 2.3.2 Phenolic Acids

Phenolic acids are a type of plant metabolite widely distributed in plant and food products.^[^ ^42^ ^]^ These compounds, which are characterized by the presence of phenolic and carboxylic acid functional groups (Figure 3), have diverse roles in plant physiology and significant health benefits in humans due to their antioxidant properties.^[^ ^43^ ^]^ Consequently, phenolic acids have received attention for the development of hydrogel electrode materials with enhanced biocompatibility and adhesion. Caffeic acid (CA) and gallic acid (GA), the most prevalent hydroxycinnamic acid derivatives, have been extensively studied for their ability to enhance hydrogel adhesion. CA can be introduced into reversible and asymmetric responsive Janus hydrogels to improve the adhesive properties.^[^ ^44^ ^]^ In addition, CA can be grafted onto injectable chitosan hydrogels to achieve high adhesion on inorganic substrates.^[^ ^45^ ^]^ Similarly, GA can be incorporated into various hydrogels to endow them with excellent adhesion.^[^ ^46^ ^]^ Under oxidative conditions, CA forms oligomeric phenol derivatives rather than high‐molecular‐weight polymers such as catecholamines. These oligomers preserve and replicate the carboxyl conjugate sites found in the monomer, making them suitable for polyphenolic modification to improve adhesive properties. Furthermore, CA/GA‐containing hydrogels have been demonstrated to have strong antioxidant and anti‐inflammatory properties. The adhesive capabilities of natural hydroxycinnamic acids other than CA and GA, including cinnamic acid, coumaric acid, rosmarinic acid, and chlorogenic acid, have not been thoroughly explored. The investigation of these acids could uncover new mechanisms and applications in the field of adhesion, potentially leading to innovations in hydrogel design and functionality, particularly for multifunctional HMI hydrogel electrodes.

#### 2.3.3 Tannins

Tannins are a diverse group of water‐soluble polyphenolic compounds found in various plants (Figure 3).^[^ ^47^ ^]^ As natural crosslinking agents, tannins have been applied to enhance the adhesive properties of hydrogels. Specifically, the multiple hydroxyl groups of tannins can form strong hydrogen bonds with both the hydrogel matrix and substrate, thereby improving adhesion.^[^ ^48^ ^]^ Tannin crosslinked hydrogels have also been found to exhibit superior mechanical strength^[^ ^49^ ^]^ and stretchability.^[^ ^50^ ^]^ Owing to their low toxicity and natural origin, tannins can improve hydrogel biocompatibility, making them particularly suitable for biomedical applications. Moreover, the anti‐inflammatory and antioxidant properties of tannins can help reduce skin irritation and enhance the overall biocompatibility of hydrogel electrodes.^[^ ^51^ ^]^

#### 2.3.4 Lignans

Lignans are naturally occurring polyphenolic compounds characterized by multiple hydroxyl groups capable of forming hydrogen bonds and other interactions with various substrates (Figure 3).^[^ ^52^ ^]^ Lignans are commonly used to enhance the adhesive properties of hydrogels via physical integration into hydrogel networks. Lignan‐based hydrogels can adhere to both hydrophilic and hydrophobic surfaces as well as biological tissues with no inflammation due to their high biocompatibility.^[^ ^53^ ^]^ In addition, lignan incorporation can improve the mechanical strength of hydrogel networks. Our group developed a tough, highly biocompatible, and plant‐inspired adhesive hydrogel electrode based on Ag‐lignin nanoparticles. The Ag‐lignin nanoparticles formed a dynamic catechol redox system, resulting in a long‐lasting reductive–oxidative environment within the hydrogel network. This redox system continuously generated catechol groups, endowing the hydrogel with long‐term and repeatable adhesiveness.^[53a]^

## 3 Tissue‐Matching Mechanical Properties for Hydrogel‐Based HMIs

**Figure 4:**
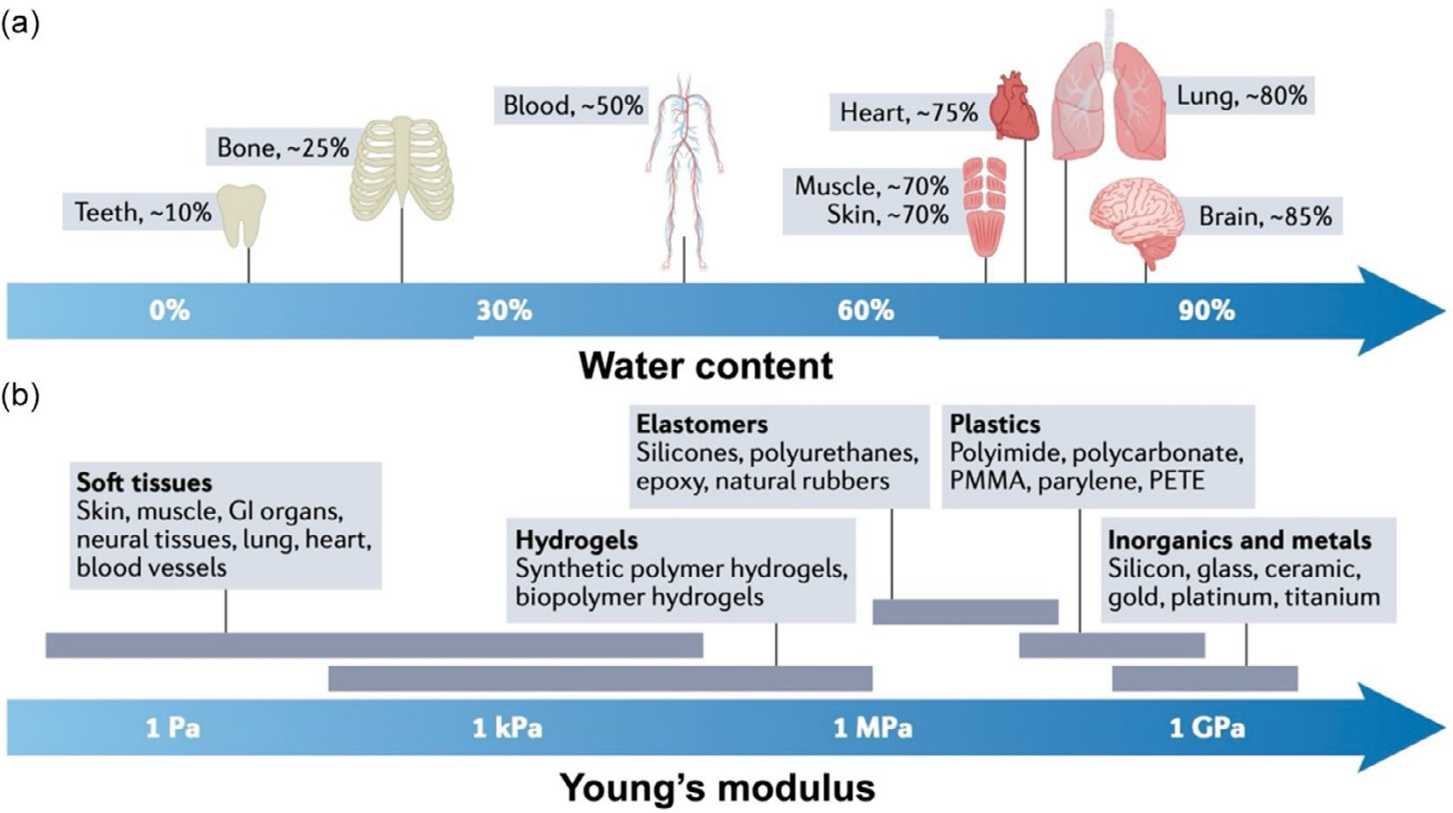
a) The water content of human tissues and b) Young's moduli comparation of soft tissues in the human between hydrogels, elastomers, plastics, inorganics, and metals. Reproduced with the permission.^[^ ^54b^ ^]^ Copyright 2022, Nature.

For hydrogel‐based HMI electrodes, mechanical properties, particularly toughness and flexibility, are critical.^[^ ^54^ ^]^ As mentioned above, most human tissues have a relatively high water content, resulting in a significantly different Young's modulus compared to traditional HMIs (**Figure** **4**). This mismatch between HMIs and human tissues can lead to discomfort and rejection. To achieve effective integration and performance, hydrogel‐based HMI electrodes must exhibit mechanical properties matching those of human tissues. This section provides an in‐depth overview of the tissue‐matching mechanical properties required to ensure that hydrogel‐based HMIs are suitable for the physiological and mechanical environments of the human body.

### 3.1 Toughness

Implantable hydrogel‐based HMIs must be tough to ensure that no damage occurs during the implantation process and that mechanical stability is maintained within the body over an extended period. To meet these implantation requirements, the mechanical strength of hydrogel‐based HMI electrodes should be sufficient to maintain structural integrity throughout the implantation process. Various strategies have been employed to improve the toughness of hydrogel electrodes. For example, the nanoenhancement effect of nanomaterials has been exploited to improve both the electrical conductivity and mechanical toughness of hydrogels. Kim et al.^[^ ^55^ ^]^ reported a TENG based on a zeolitic imidazolate framework (ZIF)‐8 nanoparticle‐reinforced conductive hydrogel for biomotion sensing and wearable HMIs. The ZIF‐8 nanoparticles improved the mechanical toughness by forming physical bonds with the polymer chains in the hydrogel network (2.7 times greater stretchability than that of pure hydrogel). Moreover, core–shell redox‐active nanoparticles were employed to enhance the toughness of the conductive hydrogel.

Bulk hydrogels can also be toughened using the double‐network strategy. Niu et al.^[^ ^56^ ^]^ presented a salt solution‐induced dual‐crosslinked dual‐network (DCDN) conductive hydrogel that exhibited excellent mechanical behavior with a toughness of 69.59 kJ m^−2^. Due to its superior mechanical properties and ionic conductivity, the DCDN hydrogel enabled the precise control of robots and prostheses. As another bulk toughening strategy, a physical crosslinking salting–freezing–thawing method was used to prepare tough hydrogel electronic skins for HMIs. Tian et al.^[^ ^57^ ^]^ reported that β‐glycerophosphate sodium (GP) induced the aggregation of poly(vinyl alcohol) (PVA) chains to prepare a tough PVA–GP/tannic acid–CaCl_2_ hydrogel via a salting–freezing–thawing strategy. This hydrogel integrated super toughness (13.96 MJ m^−3^), high electrical conductivity (4.72 S m^−1^), and strain sensitivity, making it highly suitable for future complex HMIs. High toughness is crucial to ensure reliable functionality and achieve robust integration for HMIs. Importantly, achieving hydrogel interfacial toughness primarily involves mimicking the characteristics of the target tissue, considering its unique compositional and structural features. Overall, the development of hydrogel interfaces with adjustable toughness is pivotal for future HMIs.

### 3.2 Softness for BCIs

BCIs have attracted significant attention since the first experimental demonstration in 1999 that ensembles of cortical neurons could directly control a robotic manipulator.^[^ ^58^ ^]^ BCIs are considered a potential therapy for restoring motor control in severely disabled patients, particularly those suffering from devastating conditions such as amyotrophic lateral sclerosis, spinal cord injury, stroke, and cerebral palsy.^[^ ^59^ ^]^ In BCIs, an electrode in direct contact with the brain is crucial for detecting electrical brain activity and delivering electrical stimulation.^[^ ^60^ ^]^ BCI electrodes can be classified into three categories based on their invasiveness: invasive, semi‐invasive, and noninvasive. Noninvasive electrodes, which record electroencephalograms (EEGs) from the surface of the head, offer solutions for paralyzed individuals to communicate with the outside world, although neural signals have a limited bandwidth. Both invasive and semi‐invasive electrodes are intracranial electrodes that must be placed via craniotomy. Semi‐invasive electrodes, also known as electrocorticography electrodes, are placed on the epidural or subdural surface of the cerebral cortex.^[^ ^61^ ^]^ In contrast, invasive electrodes directly penetrate soft brain tissue and record voltage potentials from a single neuron, providing high‐quality neural activity data with a wide spatial scale and remarkable anatomical diversity.^[^ ^62^ ^]^ Consequently, invasive electrodes show great potential for brain‐controlled machines. However, flexible invasive electrodes, including silicon microneedle arrays^[^ ^63^ ^]^ and platinum electrode arrays,^[^ ^64^ ^]^ still pose an infection risk, can cause bleeding, and suffer from signal deterioration during long‐term recording.

**Figure 5:**
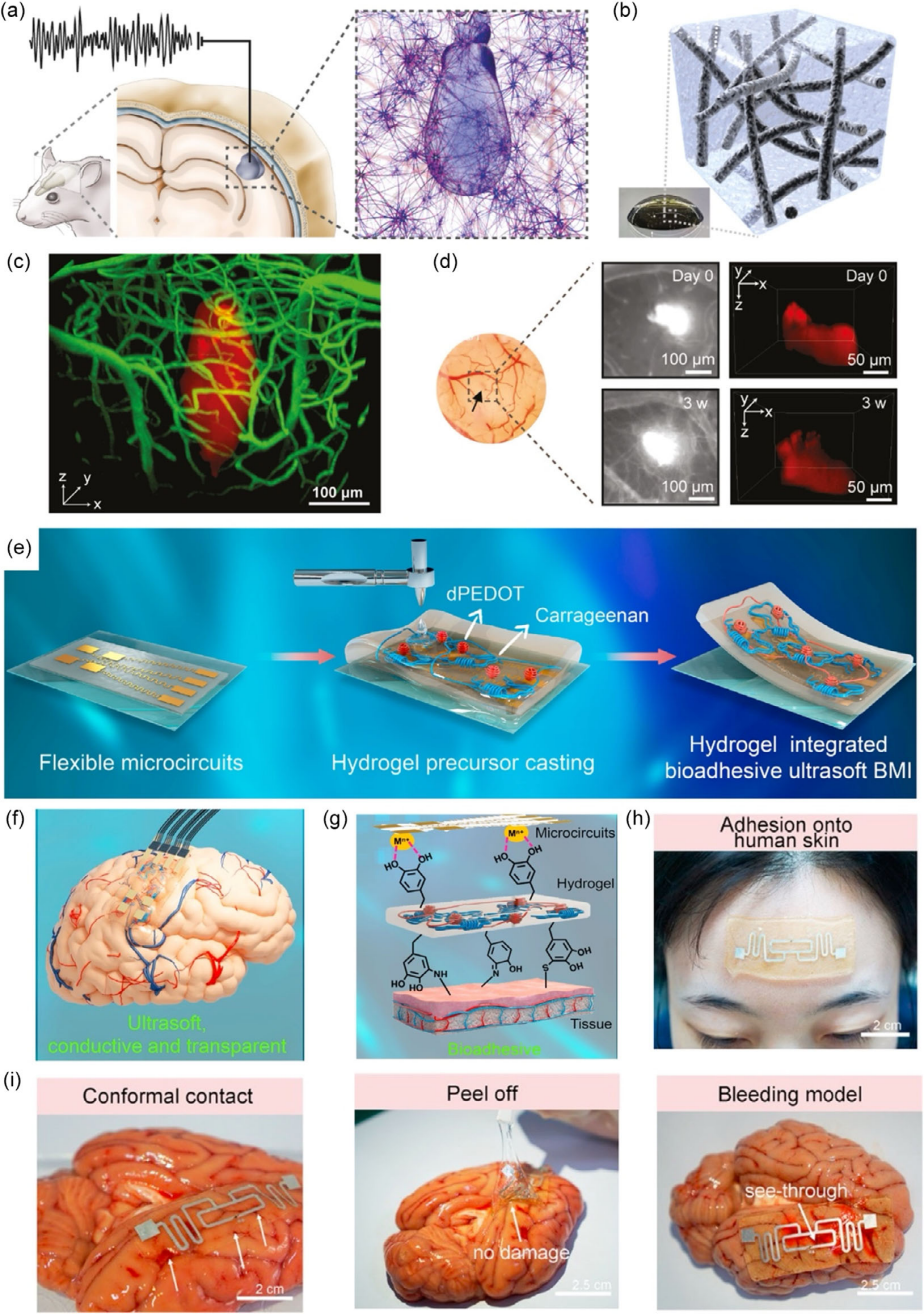
a) Schematic illustration of hydrogel injected into a mouse brain cortex and interacting with neurons, b) photograph of betaVhex/carbon nanotube (CNT) hydrogel and schematic image of the 3D electrical network of betaVhex/CNT complexes in the hydrogel, c) side view of betaVhex/CNT hydrogel injected into the brain cortex and the brain vasculature visualized via fluorescein isothiocyanate–dextran injection, and d) images of the hydrogel‐injected brain cortex on the day of injection (Day 0) and 3 weeks postinjection (3 w). Reproduced with permission.^[^ ^66^ ^]^ Copyright 2022, ACS Publications. e) Preparation process of hydrogel‐integrated bioadhesive ultrasoft BCIs, f) ultrasoft, conductive, and transparent properties of the hydrogel‐integrated BMI, g) adhesion mechanism of the bioadhesive ultrasoft BMI to soft tissue and metal substrates, h) ultrasoft hydrogel‐integrated microcircuit attached to a forehead, and i) hydrogel‐integrated microcircuit adhered to and peeled off soft porcine brain tissue, where the transparent hydrogel‐integrated microcircuit allows visualization of any tissue damage. Reproduced with permission.^[^ ^38b^ ^]^ Copyright 2021, Wiley.

Hydrogel electrodes have been recognized as alternatives to conventional electrodes because of their superior conformity to the human body. Noninvasive hydrogel electrodes with multifunctional properties, including conductivity, impedance, robustness, biocompatibility, long‐term stability, and skin adhesion, have been extensively studied.^[^ ^65^ ^]^ However, invasive hydrogel electrodes require specific features such as chemical and physical compatibility with soft neural tissues, stable conductivity, and biocompatibility to reduce procedural risks. Kim et al.^[^ ^66^ ^]^ reported a supramolecular β‐peptide‐based hydrogel electrode as a biocompatible, conductive, and biostable neural interface (**Figure** **5**a–d). This system achieved signal amplification via tight neural/hydrogel contact without neuroinflammation due to its seamless integration with brain tissue, which increased the contact area. Significant advances have also been made in the multifunctional integration of hydrogel electrodes. Our group developed a bioadhesive ultrasoft hydrogel electrode as a BCI by integrating dopamine methacrylate‐hybridized PEDOT nanoparticles into a highly conductive hydrogel. This hydrogel exhibited robust adhesiveness, enabling tight integration with metallic microcircuits and seamless adhesion to brain tissue. In addition, the hydrogel had immune‐evasive properties, which actively prevented fibrous tissue encapsulation and neuroinflammation after implantation. The as‐prepared immune‐evasive, bioadhesive, ultrasoft, and conductive hydrogel BCI was suitable for long‐term and accurate electroencephalographic signal acquisition and communication with minimal foreign body reactions (Figure 5e–i).^[38b]^ Ongoing research on hydrogel electrodes for BCIs aims to develop hydrogels that can integrate seamlessly with neural tissues while providing the mechanical and functional properties necessary for long‐term, effective operation. Miniaturizing hydrogels to control the amorphous–crystalline transition in polymers is also important for developing effective interfaces with neural tissues to facilitate the fabrication of smaller and more versatile hydrogel bioelectronics.

## 4 Electrical Performance

**Figure 6:**
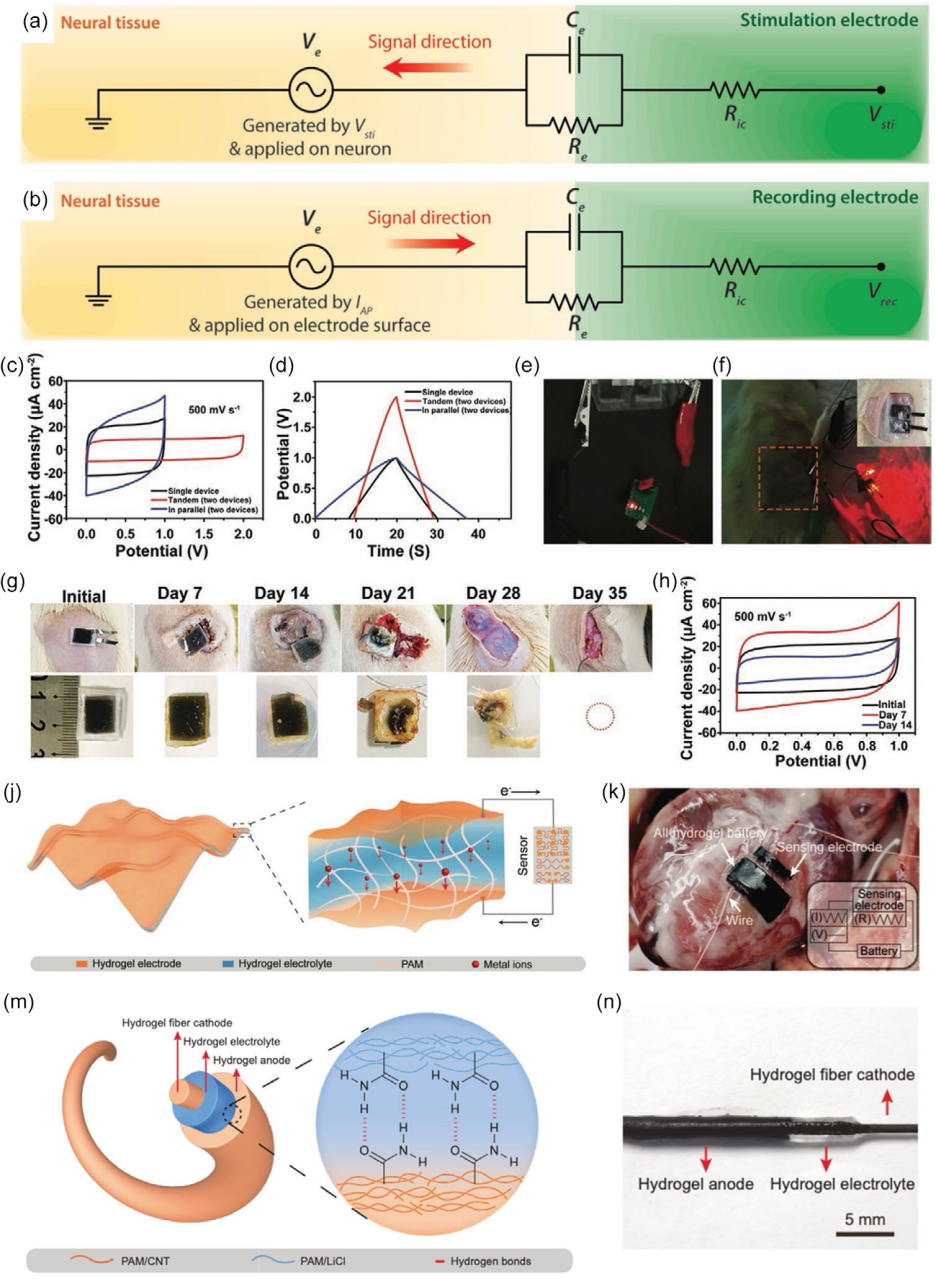
a) Equivalent circuit model of a tissue–electrode interface for bioelectronic stimulation, where *V*_sti_ represents the input for bioelectronic stimulation with interconnect resistance *R*_ic_, and *V*_e_ represents the electric potential within electrolytic media generated by the application of *V*_sti_ on the outer membrane of the targeted neurons. b) Equivalent circuit model of a tissue–electrode interface for bioelectronic recording, where *V*_rec_ represents the output for bioelectronic recording with interconnect resistance *R*_ic_, and *V*_e_ represents the electric potential within the electrolytic media generated by the neuronal bioelectronic activity (i.e., ionic current by action potentials) applied to the electrode surface. For both bioelectronic stimulation and recording, the electrolyte–electrode interface is considered as a parallel circuit of the EDL capacitance, *C*_e_, and leakage resistance, *R*_e_. Reproduced with permission.^[^ ^11^ ^]^ Copyright 2019, Royal Society of Chemistry. c) Cyclic voltammetry (CV) curves for a SrMA/A‐rGO‐based supercapacitor in single, tandem, and parallel configurations at 500 mV s^−1^. d) Galvanostatic charge–discharge curves of the supercapacitor in single, tandem, and parallel configurations at 10 μA cm^−2^. Photographs of light‐emitting diode (LED) lit by two supercapacitors in series e) in vitro and f) in vivo. Inset: photograph of device implanted subcutaneously. g) Photographs of the supercapacitor implanted subcutaneously in Sprague Dawley rats for degradation evaluation. h) CV curves for the supercapacitor after removal from the subcutaneous site at different time points. Reproduced with permission.^[^ ^82^ ^]^ Copyright 2023, Wiley. j) Schematic illustration of the structure and working mechanism of the all‐hydrogel battery. k) Detection system with power supply and sensor attached to the surface of the heart. Inset: working diagram. Reproduced with permission.^[^ ^88^ ^]^ Copyright 2023, Wiley. m) Schematic illustration and n) photograph of an all‐hydrogel aqueous lithium‐ion battery with a coaxial fiber structure. Reproduced with permission.^[^ ^90^ ^]^ Copyright 2022, Nature.

The electrical performance of hydrogel‐based HMI electrodes is a critical factor in effective bidirectional communication between machines and biological tissues.^[^ ^67^ ^]^ This communication involves the transmission of signals and information from cells to electronics and vice versa. Biological tissues, which can be considered as volume conductors with moderate conductivity in electrical models, facilitate electrical communication through ionic fluxes. In contrast, conventional electronic systems rely on electronically conductive materials, such as metals, in which free electrons act as mobile charge carriers.^[^ ^68^ ^]^ This difference creates a unique interface between the tissue and electrode, where ionically and electronically carried signals are exchanged. During bidirectional communication, electrical signals are transmitted via ionic currents and electric potentials in electrolytic tissue and by electronic currents and electric potentials in conducting electrodes.^[^ ^11^, ^69^ ^]^ The tissue–electrode interface can be depicted using an equivalent circuit model (**Figure** **6**a,b). The stimulation and recording process allows the electrolyte–electrode interface to be depicted as a parallel circuit consisting of leakage resistance *R*_e_ and electrical double‐layer (EDL) capacitance *C*_e_.^[^ ^11^ ^]^ To facilitate bidirectional communication, a low *R*_e_ and high *C*_e_ are essential.

### 4.1 Conductivity and Impedance

Improving the conductivity and reducing the impedance of hydrogel‐based HMIs is essential for enhancing bidirectional communication. Conductive polymers such as polypyrrole (PPy), polyaniline (PANI), polythiophene, and PEDOT have been used to enhance the conductivity of hydrogel‐based electrodes. These polymers can serve as network matrices for conductive hydrogels, typically resulting in high conductivity and low impedance. However, the mechanical properties of hydrogel electrodes often limit their application in wearable bioelectronics.^[^ ^70^ ^]^

Conductive polymers and their composites have also been applied as nanofillers to improve the conductivity of hydrogel electrodes with nonconductive polymer matrices and to improve the mechanical properties of conductive hydrogels. For instance, Zhou et al.^[^ ^71^ ^]^ reported hydrogels with high electrical conductivity (>11 S cm^−1^) that maintained high stretchability (>400%) and toughness (>3300 J m^−2^). To achieve high conductivity without sacrificing mechanical properties, a 3D printing process was applied to produce a bicontinuous conducting polymer hydrogel using phase‐separated inks consisting of an electrical phase (PEDOT:PSS) and a mechanical phase (hydrophilic polyurethane). Efforts have also been made to endow conductive hydrogel‐based HMI electrodes with multifunctionality. In this context, our group focused on mussel‐inspired and polyphenol‐mediated adhesives and conductive hydrogel electrodes by incorporating conductive polymer‐based composite nanofillers into a hydrogel matrix. These hydrogel electrodes exhibited high conductivity, excellent bioadhesion, and good mechanical properties, making them suitable as high‐performance hydrogel‐based HMI electrodes.[^22^, ^38^, ^39^, ^72^]

In hydrogel‐based HMIs, low impedance ensures better signal quality and efficiency, especially for the weak signals from the human body and particularly the brain.^[^ ^73^ ^]^ A high impedance may lead to signal distortion or increased noise, thereby reducing the signal‐to‐noise ratio.^[^ ^74^ ^]^ Recent studies have focused on decreasing the impedance of hydrogels by optimizing the structural and compositional properties. Some hydrogel electrodes demonstrate stable impedance across various frequencies, making them suitable for long‐term monitoring and signal acquisition in biomedical devices.^[^ ^75^ ^]^

### 4.2 Energy Storage

The energy storage properties of hydrogel‐based HMIs influence functionality, reliability, and user comfort, particularly in wearable bioelectronics or electrotherapy applications. Reliable energy storage ensures that a device can operate without frequent recharging, thereby providing uninterrupted monitoring and data collection.^[^ ^76^ ^]^ Advances focusing on improving the conductivity and reducing the impedance of hydrogel electrodes are facilitating the development of more efficient and versatile energy storage applications. The incorporation of advanced materials such as conducting polymers (e.g., PANI, PPy, and PEDOT),^[^ ^77^ ^]^ conducting nanomaterials (e.g., graphene, MXene, and CNTs),^[^ ^78^ ^]^ and nanostructures^[^ ^79^ ^]^ can significantly enhance the electrochemical properties of hydrogels. These materials provide a balance between high capacitance, conductivity, and mechanical robustness, which are essential for high‐performance energy storage devices for bioelectronics, and can inspire further studies on multifunctional hydrogel‐based HMIs.

#### 4.2.1 Supercapacitors

Supercapacitors have attracted considerable scientific interest over the past few decades due to their potential for clean energy production, easy assembly, and high performance. These advantageous characteristics have also contributed to the rapid growth of low‐power electronics, including wearable and implantable bioelectronics.[^76^, ^80^] Recently, Hu et al.^[^ ^81^ ^]^ prepared a fully flexible and omnihealable conductive PVA@PANI hydrogel electrode via an in situ freezing–polymerization strategy using an aniline/dimethyl sulfoxide emulsion template. This hydrogel electrode exhibited excellent energy storage properties due to the incorporation of pseudocapacitive PANI (936.8 F g^−1^). The as‐prepared supercapacitor conformed to a complex body surface, enabling the precise real‐time monitoring of the full range of human activities with a quick response and excellent self‐recovery. Furthermore, hydrogel electrodes with specific properties, such as biocompatibility, degradability, self‐healing, and bioadhesion, are highly attractive. For instance, a conductive, degradable, and biocompatible hydrogel electrode was prepared through the in situ polymerization of sericin commodified with aminated reduced graphene oxide and methacrylic anhydride (SrMA/A‐rGO) followed by sequential crosslinking with four‐arm polyethylene glycol succinimide carbonate and polyethylene glycol acrylate. This conductive multinetwork endowed the SrMA/A‐rGO‐based supercapacitor with good electrochemical performance, including an equivalent series resistance of 21 Ω cm^−2^, volumetric energy density of 26.0 μW cm^−2^, and high specific capacitance retention after long‐term charging/discharging. In addition to lighting LED both in vitro and in vivo, the hydrogel‐based supercapacitor served as a direct output power source to electrically stimulate a stopped heart to resume beating. Furthermore, this supercapacitor exhibited superior biocompatibility and biodegradability in vivo and maintained a specific capacitance of >30% for 2 weeks after implantation (Figure 6c–h).^[^ ^82^ ^]^

Our group has also investigated bioadhesive hydrogel‐based HMI electrodes with excellent electrochemical and mechanical properties. A series of highly bioadhesive, conductive, compressive, and stretchable hydrogel electrodes was prepared for supercapacitors. PDA‐based nanocomposite fillers, including PDA–bimetal oxide–graphene nanosheets^[^ ^83^ ^]^ and PDA–PEDOT–ZIF‐71 core–shell nanoparticles,^[^ ^84^ ^]^ were introduced to act as a mussel‐inspired redox‐active systems. When incorporated into a highly biocompatible polymer matrix, the nanofillers endowed the hydrogel with moderate adhesive and mechanical properties that matched those of human tissues, energy storage properties for supercapacitors, and in vivo biocompatibility to facilitate biosignal measurements without causing inflammation. Due to these outstanding sensing and energy storage properties, these hydrogel electrodes are highly promising candidates for next‐generation wearable self‐powered sensing electronics.

#### 4.2.2 Batteries

Batteries are the primary power sources for wearable and implantable bioelectronics because of their relatively high energy densities and stable voltage outputs. For this purpose, the mechanical properties (e.g., Young's moduli) of batteries should be similar to those of biological tissues (Figure 4).[^76^, ^85^] Unlike traditional batteries, hydrogel‐based batteries employ nontoxic and nonflammable aqueous electrolytes, which offer much higher safety.^[^ ^86^ ^]^ Hydrogel‐based batteries enable stable and close contact with uneven tissue surfaces under dynamic deformation without mechanical loading, which is beneficial for the continuous and proper functioning of bioelectronics. Moreover, matching the mechanical properties reduces physical irritation and tissue damage, thus alleviating undesirable immune responses and health hazards.^[^ ^87^ ^]^

Depending on the device structure, hydrogel‐based batteries can be divided into 2D thin‐film and 1D fiber‐shaped batteries.^[^ ^88^ ^]^ Recently, tissue‐like 2D thin‐film all‐hydrogel batteries were prepared via the dropwise addition of active material dispersions onto a PAM/CNT conductive hydrogel (Figure 6j,k). The obtained ultrasoft all‐hydrogel batteries exhibited a Young's moduli of 80 kPa, matching those of the skin and organs (e.g., the heart). Furthermore, high specific capacities of 82 and 370 mAh g^−1^ were obtained in all‐hydrogel lithium‐ion and zinc‐ion batteries, respectively, at a current density of 0.5 A g^−1^.^[87a]^ To support the development of miniaturized wearable devices, all‐hydrogel 1D batteries with coaxial or twisted structures have been explored. For instance, highly viscous polymer inks containing CNTs and either lithium iron phosphate (LFP) or lithium titanium oxide (LTO) were used to print LFP fiber cathodes and LTO fiber anodes for a flexible all‐fiber lithium‐ion battery with a twisted structure. This hydrogel‐based fiber battery can potentially be integrated into textile fabrics for wearable electronic applications.^[^ ^89^ ^]^ For applications in biocompatible and implantable bioelectronics, ultrasoft hydrogel‐based lithium‐ion batteries with a coaxial fiber structure were developed. These all‐hydrogel‐fiber‐based aqueous lithium‐ion batteries exhibited a low Young's modulus corresponding to that of biological tissues and maintained stable electrochemical performance during complex deformations (Figure 6m,n).^[^ ^90^ ^]^

These developments in the energy storage properties of hydrogel‐based HMI electrodes have facilitated advances in implantable batteries and supercapacitors. However, numerous opportunities and challenges remain, and future research should focus on developing miniaturized power devices with suitable electrochemical properties for wearable electronics, achieving mechanical properties and biocompatibility similar to those of human soft tissue to reduce irritation and foreign body reactions and incorporating novel stimuli‐responsive materials to produce self‐powering and biodegradable energy storage devices with intelligent functions.

### 4.3 Energy Harvesting

**Figure 7:**
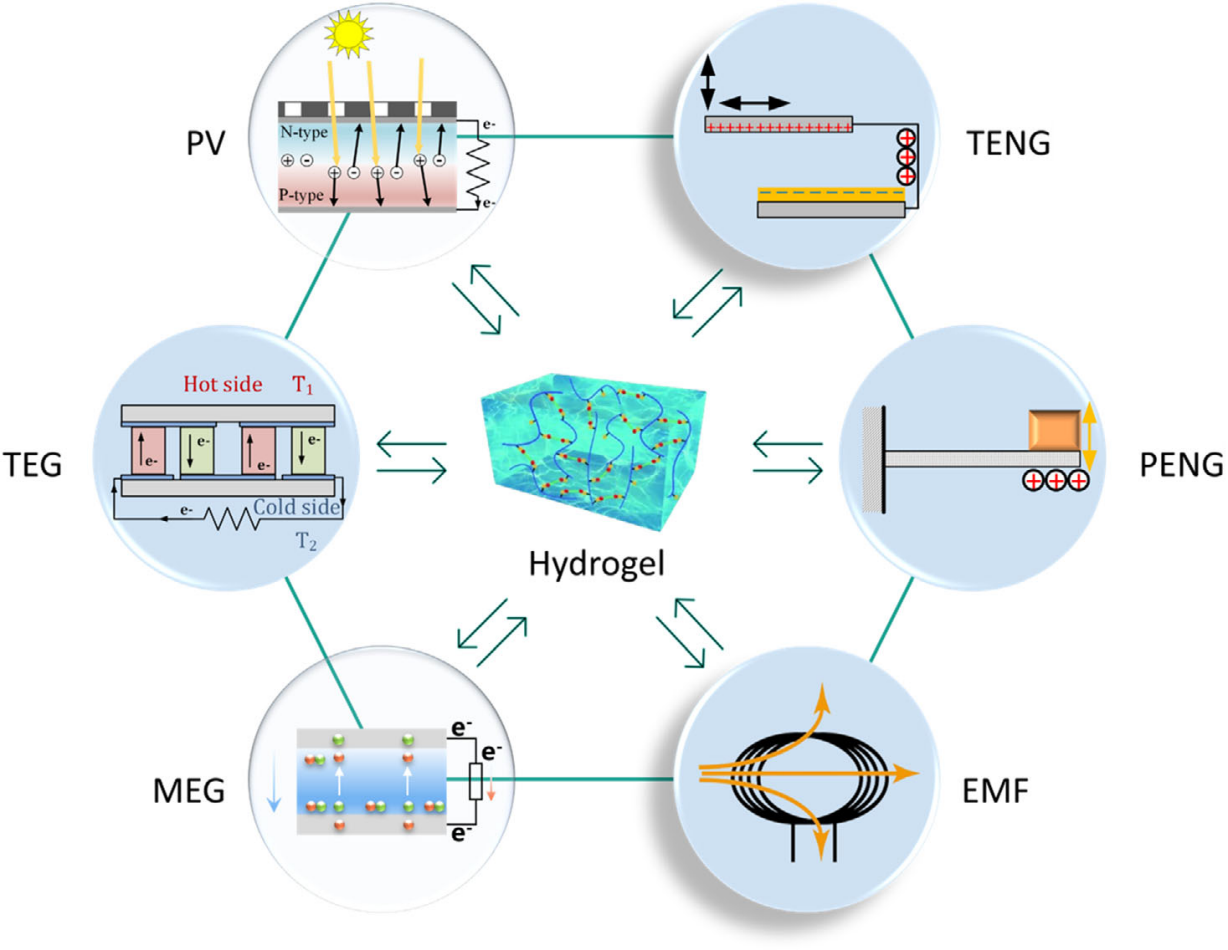
Energy harvesting technologies for hydrogel‐based bioelectronics. PV: PV energy harvesting; TEG: thermoelectric generator; MEG: moisture‐enabled electric generator; TENG: triboelectric nanogenerator; PENG: piezoelectric nanogenerator; EMF: electromagnetic field energy harvesting.

Energy harvesting technologies have emerged as promising solutions to the power supply challenges of wearable bioelectronics.^[^ ^91^ ^]^ By scavenging energy from the environment or human body, these technologies can be used to develop self‐powered, sustainable, and miniaturized devices.^[^ ^92^ ^]^ Energy harvesting technologies can also be integrated with hydrogel‐based HMI electrodes for bioelectronic applications.^[^ ^93^ ^]^ This section focuses on six categories of energy harvesting technologies for hydrogel‐based HMIs (**Figure** **7**): photovoltaic (PV) energy harvesting,^[^ ^94^ ^]^ thermoelectric generators (TEGs),^[^ ^95^ ^]^ moisture‐enabled electric generators (MEGs),^[^ ^96^ ^]^ TENGs,^[^ ^97^ ^]^ piezoelectric nanogenerators (PENGs),^[^ ^98^ ^]^ and electromagnetic field (EMF) energy harvesting.^[^ ^99^ ^]^

#### 4.3.1 Photovoltaic (PV) Energy Harvesting

PV energy harvesting involves converting light energy into electrical energy using PV cells. The integration of encapsulated and protected PV systems into hydrogel‐based HMIs can impart PV energy harvesting properties.^[^ ^100^ ^]^ Consequently, the resulting hydrogel‐based HMI electrodes are suitable for wearable bioelectronics.^[^ ^101^ ^]^ A self‐sustaining sensor based on a quasi‐2D perovskite solar cell (FPSC) was designed for continuous metabolic monitoring. Under simulated sunlight, the FPSC achieved an efficiency of 18.1%, with 16.5% efficiency for larger areas and 31.2% efficiency under indoor light conditions. This device integrates sensors to monitor the glucose levels, pH balance, sodium content, sweat production, and skin temperature. The biosensors exhibited stable performance even with low sweat flow and provided accurate results under various lighting conditions. Remarkably, this device can continuously monitor biomarkers for over 12 h without requiring a battery.^[^ ^102^ ^]^

#### 4.3.2 Thermoelectric Generators (TEGs)

TEGs operate based on the Seebeck effect, where a voltage is generated when a temperature difference exists between two dissimilar materials.^[^ ^103^ ^]^ In TEGs integrated with hydrogels, the hydrogel serves as a flexible heat sink to dissipate heat via water evaporation and create a temperature gradient. These gradient drives charge carrier movement within the thermoelectric material, thereby generating electrical power. Hydrogel‐based TEGs attached to human skin can harvest body heat, providing a continuous and sustainable power source for wearable devices.^[^ ^104^ ^]^ The device is a body‐heat‐powered wearable health monitoring solution. It operates by generating sufficient power from a small temperature difference of 2–4 K to operate the sensors and Bluetooth modules. Integrated energy management electronics boost low voltages (30 mV) to usable levels (3.3–5 V). Featuring highly flexible and stress‐resilient electrode configurations, the device achieved effective wireless transmission cycles as short as 1.6 s, ensuring reliable performance under wearable conditions.

#### 4.3.3 Moisture‐Enabled Electric Generators (MEGs)

MEGs, which use variations in moisture to generate electricity, can be categorized as evaporation‐induced generators (EIGs) or moisture‐induced generators (MIGs).^[^ ^105^ ^]^ EIGs harness energy from water evaporation, whereas MIGs rely on moisture flow and proton diffusion. Because of their high water absorption and retention capacities, hydrogels play a crucial role in MEGs by providing a continuous water supply for evaporation and facilitating proton transport.^[^ ^106^ ^]^ Integrating hydrogel‐based HMI electrodes with MEGs is a promising approach for harvesting energy from ambient humidity or bodily fluids, such as sweat. The state‐of‐the‐art MEG achieves an output current density of 0.625 mA cm^−2^ at 90% relative humidity, maintaining 90% of its performance after 1 month and generating over 3 W m^−2^. In addition, a wearable sweat‐driven system incorporated Bluetooth wireless transmission and achieved a reliable sampling frequency of 100 Hz.^[^ ^107^ ^]^

#### 4.3.4 Triboelectric Nanogenerators (TENGs)

The operation of TENGs is based on triboelectric effect and electrostatic induction. The triboelectric phenomenon occurs when two dissimilar materials come into contact and then separate, thereby generating static charges.^[^ ^108^ ^]^ Hydrogels can be integrated into TENGs as triboelectric layers or as substrates for triboelectric materials.^[^ ^55^, ^109^ ^]^ Because of the flexibility and stretchability of hydrogels, TENGs can conform to the human body and capture energy from various body movements, including walking, running, and even subtle gestures.^[^ ^110^ ^]^ TENGs exhibit power densities ranging from 138 to 3.8 mW cm^−2^, with various sizes including 60 × 78 mm for fabric‐based systems and 50 × 75 mm for backpack‐integrated designs. These devices are used primarily for electronic charging, illumination, and biomotive energy harvesting. Their connection to the body can be facilitated through tape, fabric, or backpacks, enabling TENGs to integration into wearable and portable energy‐harvesting systems for a variety of applications.

#### 4.3.5 Piezoelectric Nanogenerators (PENGs)

PENGs rely on the piezoelectric effect, in which an electrical charge is generated in response to mechanical stress on certain materials.^[^ ^111^ ^]^ Hydrogels with inherent piezoelectric properties or those incorporating piezoelectric materials can effectively convert mechanical energy from body movements or muscle contractions into electrical energy.^[^ ^112^ ^]^ PENGs offer a versatile energy‐harvesting solution for wearable bioelectronics because they can be integrated into clothing, footwear, and implanted devices.^[^ ^113^ ^]^ PENGs achieve power densities ranging from 0.6 μW cm^2^ to 667.2 μW cm^−3^, depending on the materials utilized. Their dimensions vary; examples include PVDF‐g‐MA‐fabric‐based nanogenerators and glycine‐PVA biodegradable films measuring 10 × 10 mm. These devices connect to the body using fabric or biodegradable films and are applied in areas such as wearable electronics, implants, and sign language interpretation using flexible electronic skins.

#### 4.3.6 Electromagnetic Field (EMF) Energy Harvesting

EMF energy harvesting involves capturing energy from ambient electromagnetic waves, such as radio frequency (RF) signals.^[^ ^114^ ^]^ Hydrogels can be used to fabricate flexible antennas that can harvest RF energy and transmit data wirelessly.^[^ ^115^ ^]^ As the conformal adhesion of hydrogels to tissue surfaces enhances the efficiency of wireless power transfer, these systems are suitable for use in implantable bioelectronics. The cutting‐edge wearable hydrogel utilizing wireless power transfer features an MXene‐based 5G antenna energy harvester with a power density of 0.005 mW cm^−2^, operating at 915 MHz.^[99b]^ This system generates ≈160 μW and maintains a power transfer efficiency of 99% even when bent at a 90° angle, making it well suited for flexible, wearable applications.

These energy‐harvesting technologies provide inspiration for further developments in hydrogel‐based HMI electrodes for bioelectronic applications, particularly implantable devices. Harnessing the kinetic and mechanical energy generated by human body movements ensures long‐term operation and stability of implanted devices while reducing user inconvenience. However, integrating these energy‐harvesting technologies into hydrogel‐based HMIs without compromising other performance characteristics remains a challenge to be addressed in further work. Additionally, a summary of the various performance metrics of the polyphenol‐mediated multifunctional HMI hydrogel electrodes and their applications is presented in **Table** **1**.

**Table 1:** Summary of the performance and applications of polyphenol‐mediated multifunctional HMI hydrogel electrodes.

Polyphenol	Hydrogel network	Properties				Applicationa)	References
		Adhesion	Conductivity	Mechanical	Impedance		
Levodopamine	PAA/CS‐	23.9 kPa on pig skin	0.88 S m^−1^	Tensile strain (≈1100%) stress (164 kPa)	–	Sensor GF: 1.14 (<300%), 2.92 (300–700%), 7.34 (700–850%), and 25.18 (850–970%)	[157]
3,4‐dihydroxy benzaldehyde	PVA	–	13.48 mS cm^−1^	Tensile stress (6.47 MPa), tensile strain (1000%), toughness (35.24 MJ m^−3^) modulus (2.44 MPa)	–	TENG open circuit voltage (≈150 V), short‐circuit (≈3.0 μA)	[158]
GA/Fe3+	PAM	55 kPa on pork skin	100 mS m^−1^	Tensile strength (≈470 kPa) elongation ratio (≈920%)	–	Sensor GF: 2.47 (0–100%), 3.92 (100–150%), and 5.54 (150–200%)	[159]
Lignin	PAM	wood (0.77 MPa), iron (0.63 MPa) rubber, (0.46 MPa), PET (0.36 MPa).	1.22 mS cm^−1^ (ionic conductivity)	High stretchability (640%)	–	TENG voltage output of 265 V and a consistent current of 2.7 μA within a confined 2 × 3 cm^2^ area	[160]
Tea polyphenols	CS/PAA	Wood (9.41 kPa), iron (11.34 kPa), glass (7.80 kPa) PTFE (3.83 kPa)	6.67 S m^−1^	Tensile stress (0.075 MPa) tensile strain (1184%), toughness (0.48 MJ m^−3^)	–	Sensor GF: ≈1.7 (600%)	[161]
Gallic acid/polydopamin	PEDOT/PGA/GelMA	Wet adhesive strength (13 kPa) on porcine hearts	>15 S m^−1^	Biodegradable: lost 90% of their weight in 21 d (addition of 2 μg mL^−1^ in vivo)	–	Myocardial infarction repair	[162]
Tannic acid	PAM/glycerol/gelatin/tannic acid/Fe^3+^	98 kPa on pigskin	1.0 mS cm^−1^	–	Interfacial impedance is 650.79 Ω cm^2^ (frequency of 10 Hz)	Sensor GF: 2.314 sSNR = 20.21 dB	[163]
Tannic acid	TA/CNF/PAM	–	43.6 mS m^−1^	Strength (156.4 kPa), stretchability (1624.8%)	–	Electronic skin sensor	[164]
Polydopamine	PAM/PDA	20.20 kPa on tissue	0.41 mS cm^−1^	Fracture strength (76.22 kPa), toughness (137.44 kJ m^−3^), Young's modulus, (27.11 kPa)	–	Sensor GF: 0.497 Supercapacitor pecific capacitance (108.37 mF cm^−2^ at a current density of 1 mA cm^−2^)	[165]
Tannic acid	TA/PANI/PAAm	2.9 Kpa on paper	23.4 S m^−1^	Tensile strain (903%)	–	Supercapacitors capacitance (570 mF cm^−3^ at 1 mA cm^−2^) volume energy density (55.9 μWh cm^−3^), capacitance retention (85.6 % after 2000 cycles).	[166]
Polydopamine	PEDOT@PZIF‐71/PAM	37 kPa on pigskin	50.2 S m^−1^	Tensile strength (2200%), tensile strain (46 kPa) fracture energy (4200 J m^−2^) compressive stress (300 kPa at a strain of 80%)	–	Supercapacitor Capacitance (408.4 mF g^−1^ at a current density of 1.6 mA g^−1^) retention (84.9% after 100 cycles)	[84]
Polydopamine	PAM/PrGO/CNON	10.6 kPa on pigskin	–	Tensile strength (90 kPa) tensile strain (1700%), compressive stress (300 kPa, 80% strain)	–	Supercapacitor Capacitance (256.63 F g^−1^ /1.16 mF cm^−3^) energy density (126.64 Wh kg^−1^), power density (75.98 kWh kg^−1^) capacitance retention (91.3%, 1000 cycles)	[83]
Tannic acid	PVA/gellant gum/TA/borax	4.62 kPa to pigskin	–	Stretchability (>1200%), toughness (268.6 kJ m^3^)	8.3 kΩ at 1 kHz	I‐skin GF: 0.26 (in the strain ranges of 0–200%)	[167]
Tannic acid	TA@WSCA/PAA/ SBMA	plastic (1.14 MPa), glass (0.95 MPa), wood (1.01 MPa)	–	Tensile strength (1.33 MPa) and toughness (4.89 MJ m^−3^).	–	Electrolyte for supercapacitor Capacitance (241.5 F g^−1^ current density is 0.5 A g^−1^), retention (90% for 10 000 cycles)	[168]
Tannic acid	PAM	Zinc foils surface (49.5 kPa)	32.91 mS cm^−1^ (ion conductivity)	Tensile strength (34.95 kPa) fracture elongation (201%).	–	Electrolyte for the Zn//Zn symmetric cell (cycling stability 0f 2000 h at 0.5 mA cm^−2^).	[169]
Hydroxy	PEDOT:PSS/PAM@SA	glass (≈65 kPa)	–	Compressive modulus (≈20 kPa)	Wet hydrogel (<0.4 kΩ), dry electrodes (≈15 kΩ)	Noninvasive BCIs	[65a]
Dopamine	CA/PDA/PAM/dPEDOT NPs	Rat's brain (10 kPa)	42.0 ± 0.6 S m^−1^	Dynamic modulus (1.0 kPa) tensile strain (2500%), fracture energy (1.35 ± 0.08 kJ m^−2^)	–	BMI for epidermal brain signal detection SNR = 10.19 dB	[38b]
Tannic acid	MXene/HA/PBA/TA	≈10.17 kPa on Porcine skin,	≈1.8 × 10^−4^ S cm^−1^	Modulus (≈1.2 kPa)	–	EMG signal detection	[170]
Tannic acid	Li/AA/TA/PAM	296.875 kPa on aluminum foil	1.13 S m^−1^	–	–	Thermoelectric Generators the operation temperature of commercial polycrystalline silicon solar cells was reduced by 16 °C under an illumination of 1 kW m^−2^, and the corresponding efficiency of energy conversion was increased by 1.14%	[171]
Dopamine	PAM/PAA	Wet adhesion ≈20 kPa on tissue	≈10 S m^−1^	Tensile strength (≈60 kPa), tensile strain (1600%), modulus (≈3.9 kPa)	–	Immunomodulation, Wound Healing, and in vivo epicardial ECG signal detection	[72a]
Lignin	Poly(acrylamide*‐co*‐[2‐(methacryloyloxy)ethyl]dimethyl‐(3‐sulfo‐propyl) ammonium hydroxide)	0.248 mN m^−1^	–	–	≈10^5^ Ω cm^2^, frequency of 10 Hz	EMG signals monitor SNR = 44.4 dB	[172]
Lignosulfonate	PAM	Interfacial toughness (240 J m^−2^) on porcine skin	3.87 × 10^−2^ S cm^−1^	Elongation (600%) modulus (25 kPa)	5.2 × 10^3^ Ω at 1000 Hz	ECG and EMG signals monitor SNR ≈ 34.55 dB	[173]
Polydopamine	PAM	20 kPa on porcine skin	108 S m^−1^	Tensile stress (90 kPa) tensile strain (2000%), fracture energy (3750 J m^−2^)	–	EMG, EEG, and ECG signals monitor	[39]
Polydopamine	Gelatin	(≈0.02 kPa^−1^) on human skin	–	Tensile strength (≈160 kPa), tensile strain (75%), Young's modulus (≈55 kPa)	≈50 KΩ	EMG signals monitor SNR ≈ 32.04 dB	[174]
Polydopamine	Carboxymethyl cellulose	porcine skin (93.95 J m^−2^)	0.58 S m^−1^	Tensile strain (88–159%) elastic modulus (0.914 MPa)	–	ECG and EMG recording and FES SNR ≈ 15 dB	[148]

a) GF, gauge factor; TENG, triboelectric nanogenerator; PAM, polyacrylamide; PAA, polyacrylic acid; GelMA, d‐gelatin methacrylamide; SNR, signal‐to‐noise ratio; TA, tannic acid; PDA, polydopamine; PANI, polyaniline; CA, carrageenan; PEDOT, hybridized poly(3,4‐ethylenedioxythiophene); ECG, electrocardiogram; EMG, electromyogram; EEG, electroencephalogram; FES, electrical stimulation.

## 5 Biocompatibility

Biocompatibility is crucial for the long‐term safety and effectiveness of hydrogel‐based HMIs, as it minimizes adverse immune responses, reduces inflammation, and ensures reliable integration with biological tissues for accurate signal transmission and sensor performance.^[^ ^116^ ^]^ The biocompatibility of hydrogel‐based HMIs is largely dependent on the polymer matrix of the hydrogel and the incorporated nanofillers. Hydrogel matrices commonly consist of naturally derived polymers, such as collagen, hyaluronic acid, chitosan, gelatin, alginate, agarose, carrageenan, pectin, and dextran, because of their inherent biocompatibility and biological activity. These polymers support cell growth and tissue integration, making them suitable for biomedical applications.^[^ ^117^ ^]^ Such hydrogel electrodes can be combined with various nanofillers that impart additional functionalities such as conductivity, self‐healing capabilities, antibacterial properties, and biodegradability. However, natural polymers have inherent limitations, including mechanical weakness, modification difficulties, poor stability, and a tendency to induce immunogenic responses, which can hinder their broader application in hydrogel‐based HMIs.^[^ ^118^ ^]^

In contrast, hydrogels derived from synthetic polymers such as poly (ethylene glycol) (PEG), PVA, polyoxyethylene, polylactic acid, polycaprolactone, and PAM have adjustable microstructures, enhanced durability, and high mechanical strength. However, these polymers lack biological activity. Naturally derived hydrogels are more favorable than synthetic hydrogels for controlling cell adhesion, migration, differentiation, and growth. To integrate the advantages of both natural and synthetic polymers, numerous hydrogel electrodes have been prepared using the double‐network strategy.^[54b]^

Additionally, nanofillers or inorganic ions with suitable biocompatibility can be integrated to ensure the functionality of hydrogel electrodes. For example, biomass‐derived phenolic compounds can endow hydrogels with bioadhesion. Furthermore, conductive polymers, graphene, MXene, zwitterions, and CNTs can enhance the conductivity of hydrogel electrodes. In addition, growth factors can influence cell behavior. Thus, organic–inorganic hybridization has become an efficient strategy for synthesizing next‐generation hydrogel‐based HMIs.

## 6 Biofouling Resistance

Biofouling, which is the undesirable accumulation of microorganisms, cells, or biomolecules on surfaces, presents a significant challenge in the field of bioelectronics,^[^ ^119^ ^]^ particularly for implantable or wearable devices. This phenomenon can impair the functionality, longevity, and biocompatibility of hydrogel‐based HMI electrodes.^[^ ^120^ ^]^ Various strategies have been explored for mitigating biofouling in hydrogel‐based HMIs. First, the surface of a hydrogel‐based HMI electrode can be modified with antifouling coatings or functional groups to discourage the attachment of biofouling agents. Zwitterionic polymers such as PEG and hydrophilic polymers form hydration layers that prevent protein adsorption and bacterial adhesion.^[^ ^121^ ^]^ Second, the incorporation of antibacterial agents, such as silver nanoparticles, antibiotics, or quaternary ammonium compounds, into the hydrogel matrix can inhibit bacterial growth and colonization.^[^ ^122^ ^]^ Third, designing hydrogel‐based HMI electrodes that are responsive to external stimuli, such as pH or temperature changes, can trigger the release of antifouling agents or alter surface properties to prevent biofouling.^[^ ^123^ ^]^ Finally, modifying the surface topography of hydrogel‐based HMI electrodes by creating micro‐ or nanoscale patterns can disrupt the attachment and growth of microorganisms.^[^ ^124^ ^]^

Thus, hydrogels require properties such as antibacterial activity and surface modification potential to address biofouling challenges. For example, the incorporation of antibacterial additives, such as silver nanoparticles, or surface modification with catechol‐based derivatives can enhance the resistance of hydrogels to biofouling.^[^ ^124^ ^]^ Further research is necessary to develop comprehensive antibiofouling strategies for hydrogels that consider the intended application and nature of the biofouling agents. The development of biofouling‐resistant hydrogels is crucial for the long‐term success and widespread adoption of hydrogel‐based bioelectronics in various biomedical applications.

## 7 Stability under Physiological Conditions

Hydrogel stability under physiological conditions is critical for developing bioelectronics, especially for in vivo or on‐body applications. Physiological conditions present a complex environment, in which the pH, temperature, ionic strength, and presence of enzymes and biomolecules can vary.^[^ ^125^ ^]^ Hydrogel‐based HMI electrodes can degrade or lose structural integrity under these conditions due to pH disruption, enzymatic degradation, or the effects of ionic strength on electrostatic interactions.^[^ ^126^ ^]^

Several strategies can enhance the stability of hydrogel‐based HMI electrodes under physiological conditions.^[^ ^127^ ^]^ For example, increasing the degree of chemical crosslinking within the hydrogel network, particularly via the double‐network strategy, can improve the degradation and swelling resistance.^[^ ^128^ ^]^ Moreover, selecting polymers with inherent stability under physiological conditions, such as PEG or PVA, can enhance hydrogel longevity. In addition, coating the hydrogel surface with protective layers or modifying it with functional groups can provide protection against enzymatic degradation and pH fluctuations. Incorporating stabilizing agents, such as antioxidants or enzyme inhibitors, can protect the hydrogel from oxidative damage or enzymatic degradation. Furthermore, the addition of inorganic salts^[^ ^129^ ^]^ or cryoprotectants^[^ ^105^ ^]^ can improve the resistance of the hydrogel to freezing or dehydration. However, because of the diverse and complex fluid environments encountered by hydrogel‐based HMIs, further efforts and innovations are required to systematically address stability issues and tailor enhancement strategies for specific human tissues and applications.

## 8 Anti‐Inflammatory Properties

The inflammatory response is a natural defense mechanism of the body against injury or infection. However, excessive or chronic inflammation can lead to tissue damage and various diseases, highlighting the importance of imparting hydrogel‐based HMI electrodes with anti‐inflammatory properties.^[^ ^130^ ^]^

Several strategies have been explored to introduce anti‐inflammatory properties into hydrogel‐based HMI electrodes.^[^ ^131^ ^]^ The anti‐inflammatory properties of hydrogel‐based HMI electrodes can be improved by incorporating anti‐inflammatory drugs. Drugs such as corticosteroids or nonsteroidal drugs can be released in a controlled manner at the inflammation site. The delivery of therapeutic cells (mesenchymal stem cells), biomolecules (cytokines), or growth factors through hydrogel‐based HMI electrodes, which serve as carriers, can modulate the immune response and promote tissue repair.^[^ ^132^ ^]^ Moreover, as matrices for hydrogel‐based HMI electrodes, some natural polymers, such as hyaluronic acid and chitosan, have inherent anti‐inflammatory properties due to their ability to interact with immune cells or scavenge reactive oxygen species (ROS).^[^ ^130^ ^]^ Efforts have also been made to synthesize stimuli‐responsive hydrogel‐based HMI electrodes.^[^ ^133^ ^]^ Such systems can respond to inflammatory stimuli such as changes in pH or the presence of inflammatory markers, release anti‐inflammatory agents, or alter their properties to enhance therapeutic effects.

Effective anti‐inflammatory hydrogels are promising for treating inflammatory diseases and promoting tissue regeneration. However, further research is necessary to optimize the design and functionality of anti‐inflammatory hydrogels and to tailor them for specific inflammatory conditions and therapeutic goals.

## 9 Antioxidant Properties

Oxidative stress plays a critical role in the adhesion between hydrogel electrodes and target tissues.^[54b]^ Oxidative damage in the surrounding tissues can promote protein and cell adhesion to hydrogel electrodes,^[^ ^134^ ^]^ which increases the interfacial resistance and ultimately impairs the function of HMI electrodes. Therefore, hydrogel‐based HMI electrodes require antioxidant capabilities to modulate oxidative stress and ensure in vivo stability.

Polyphenol‐mediated hydrogels, which are enriched with antioxidant molecules, can effectively mitigate oxidative stress by neutralizing ROS.^[^ ^135^ ^]^ This approach not only protects the hydrogel electrode but also limits damage in the surrounding biological tissues, thereby enhancing the overall device performance and durability. The mechanism of action involves electron donation to ROS to produce less reactive and harmful species.^[^ ^136^ ^]^

The antioxidant properties of polyphenol‐mediated hydrogels significantly improve the durability and functionality of bioelectronic devices. By preventing oxidative degradation, these hydrogels maintain their structural integrity and performance over extended periods, which is beneficial for long‐term implants and wearable devices exposed to physiological conditions prone to inducing oxidative stress.

## 10 Promising Applications in Bioelectronics

**Figure 8:**
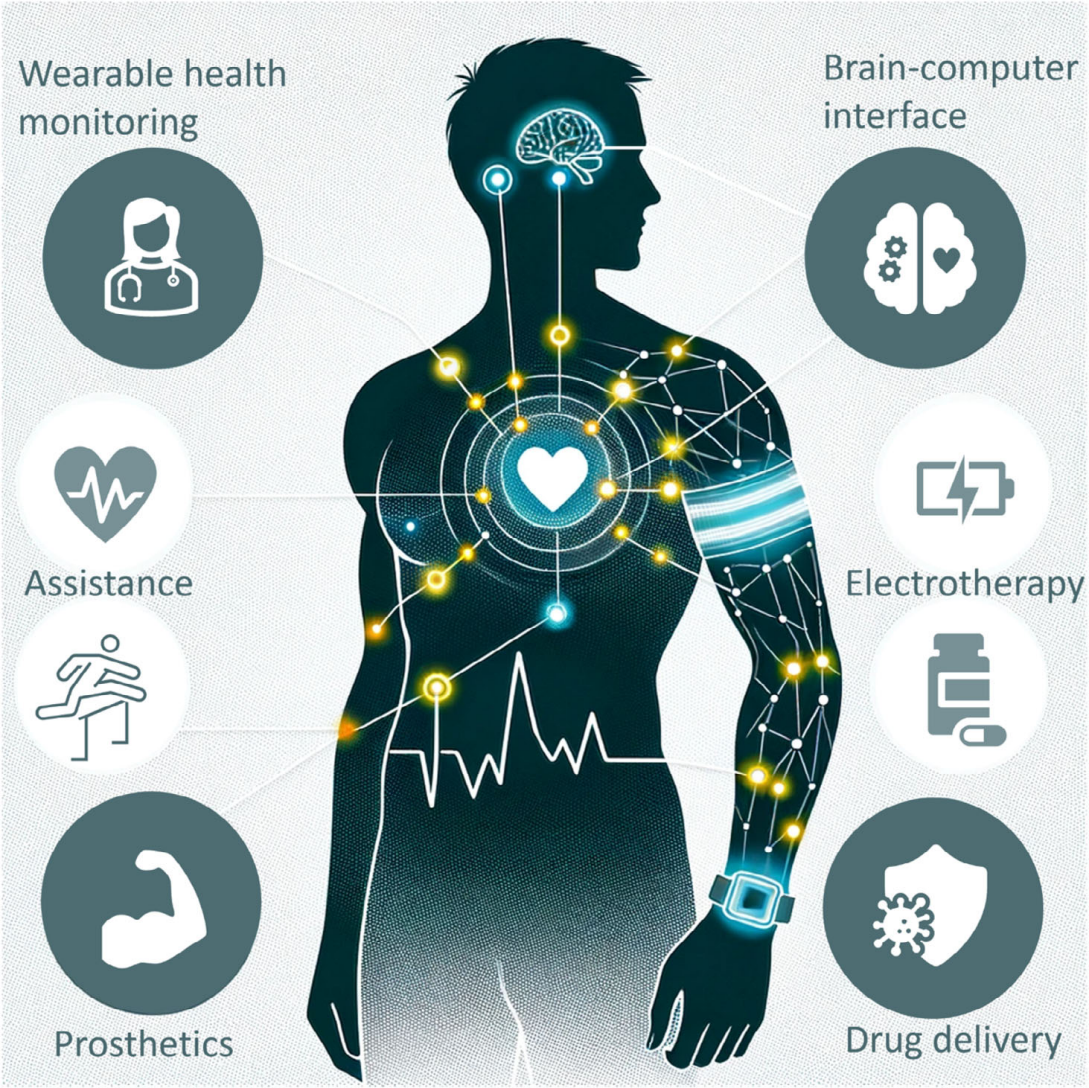
Potential bioelectronic applications of polyphenol‐mediated and mussel‐inspired hydrogel‐based HMI electrodes.

Polyphenol‐mediated multifunctional hydrogel‐based HMI electrodes have emerged as a promising technology for bioelectronics because of their various advantageous characteristics, as described previously. Considerable efforts have been devoted to the use of these hydrogel electrodes in bioelectronics, and key applications are discussed in this section (**Figure** **8**).

### 10.1 Neural Interfacing and BCIs

In neural interfacing applications, polyphenol‐mediated HMI hydrogel electrodes can be used for the high‐fidelity recording of neural signals. Their robust adhesion and high conductivity enable precise and stable signal acquisition from the brain, which is crucial for understanding neural activity and diagnosing neurological disorders.^[^ ^137^ ^]^ These hydrogel electrodes can also deliver accurate electrical stimulation to specific neural tissues, offering therapeutic benefits for Parkinson's disease, epilepsy, and chronic pain.^[^ ^138^ ^]^ Furthermore, owing to their excellent biocompatibility and flexibility, polyphenol‐mediated HMI hydrogel electrodes have potential for use in implantable neural devices. Due to their tissue‐matching mechanical properties and bioadhesion, these devices can interact directly with neural tissues, providing both recording and stimulation functions for advanced neural prosthetics and therapeutic interventions.

In BCIs, polyphenol‐mediated hydrogel electrodes can be used to acquire high‐fidelity EEG signals. The strong skin adhesion and flexibility of these electrodes ensure comfort and stability during prolonged use, enhancing the user experience and accuracy for communication, control, and gaming applications.^[^ ^139^ ^]^ For high‐resolution brain signal acquisition and stimulation, hydrogel electrodes can be implanted to establish a direct interface with brain tissues.^[38b]^ Such accurate brain activity recordings can facilitate the detection of early signs of mental health conditions, including depression or anxiety, enabling timely interventions. Furthermore, assistive technologies play a crucial role in restoring motor function in individuals with paralysis or other motor impairments by decoding brain signals to control external devices, such as prosthetics or exoskeletons.^[^ ^140^ ^]^

### 10.2 Wearable Health Monitoring and Medical Diagnostic Devices

Polyphenol‐mediated multifunctional hydrogel‐based HMI electrodes have been extensively applied in wearable devices for monitoring physiological signals, such as heart rate, muscle activity, and brain waves.^[^ ^138^, ^141^ ^]^ Their strong adhesion ensures stable skin contact, facilitating accurate and reliable data collection. Due to their high conductivity and low impedance, these electrodes can also provide high‐quality signal acquisition for electrocardiograms, EEGs, and other health‐monitoring applications. This ability is crucial for diagnosing and monitoring cardiovascular and neurological conditions.^[139b]^ Additionally, the hydrogel‐based HMI electrodes can contribute to the management of chronic diseases, monitoring sleep patterns, assessing respiratory functions, and offering real‐time data for optimized treatment plans and early intervention.^[^ ^142^ ^]^ When integrated into smart bandages for wound healing, these systems play roles in monitoring healing progress, preventing infections, and enhancing recovery conditions.^[^ ^143^ ^]^ These advances underscore the pivotal role of polyphenol‐mediated HMI hydrogel electrodes in advancing healthcare technology.

### 10.3 Prosthetics and Assistive Technologies

In the field of prosthetics and assistive technologies, polyphenol‐mediated HMI hydrogel electrodes can enhance the effectiveness and responsiveness of neural interfaces, particularly in advanced prosthetic limbs.^[^ ^144^ ^]^ By enabling stable and high‐fidelity neural signal acquisition and precise stimulation, which are crucial for achieving better control and feedback in prosthetics, these electrodes can enhance user capabilities in daily activities. Moreover, the impact of the hydrogel electrodes can be extended to exoskeletons, electronic skin, and soft robotics. Their mechanical properties and ability to form strong conformal contacts with biological tissues make these electrodes ideal for improved muscle activity monitoring and electrical stimulation. Due to this versatility, polyphenol‐mediated hydrogel electrodes are pivotal components in advancing the functionality of prosthetic and assistive technologies.

### 10.4 Electrotherapy

The enhanced energy storage and energy harvesting capabilities of polyphenol‐mediated multifunctional hydrogel electrodes offer promising applications beyond the electrical stimulation of neural tissues. Electrode integration enables the targeted delivery of electrical stimulation to specific body parts, particularly in implantable electrotherapy. This ability, which is valuable for treating conditions such as chronic pain and inflammation, can also facilitate electrotherapy for pain management and muscle rehabilitation. By delivering precise electrical impulses to the target tissues, these electrodes contribute to pain relief, muscle strengthening, and accelerated recovery from injuries. Furthermore, hydrogel electrodes are suitable for incorporation into tissue engineering and regenerative medicine strategies, where controlled electrical stimulation can support tissue growth, repair, and regeneration. This multifaceted approach emphasizes the potential of polyphenol‐mediated hydrogel electrodes for advancing therapeutic interventions and biomedical applications.

### 10.5 Drug Delivery Systems

Polyphenol‐mediated hydrogel electrodes can be incorporated into smart drug delivery systems, in which controlled drug release occurs in response to electrical signals. Such precise and targeted drug delivery can improve therapeutic outcomes.^[^ ^145^ ^]^ Furthermore, the porous electrode structure facilitates the loading of various drugs, ranging from small molecules to proteins and nucleic acids. The drug‐release kinetics can be tailored by modifying the hydrogel composition, crosslinking density, and degradation rate. Stimuli‐responsive hydrogels can further enhance drug delivery by responding to specific triggers, such as pH or temperature changes, and releasing the drug at the desired location and time.^[^ ^123^ ^]^ This targeted and controlled drug delivery approach has potential applicability in cancer therapy, wound healing, and the treatment of chronic diseases.

In summary, due to their polyphenol‐mediated multifunctionality, HMI hydrogel electrodes are ideal for developing flexible and stretchable electronic devices. By conforming to different body shapes and movements, these devices enhance comfort and usability. Furthermore, these characteristics make the polyphenol‐mediated HMI hydrogel electrodes suitable for a wide range of bioelectronic applications, pushing the boundaries of current technologies in health monitoring and neural interfaces.

## 11 Challenges and Perspectives

Despite their promising applications, polyphenol‐mediated multifunctional HMI hydrogel electrodes present several challenges for bioelectronics, as summarized below. 1) The long‐term stability and durability of hydrogels in biological environments are major issues. Over time, exposure to bodily fluids can lead to electrode degradation and the accumulation of proteins, cells, and other biomolecules on the electrode surface, potentially affecting the structure and adhesion performance. Additionally, mechanical stress can compromise stability and device performance. Managing the immune response to any foreign material and understanding and controlling the degradation mechanisms of hydrogel‐based HMI electrodes during long‐term implantation in the body are essential to prevent inflammation or rejection, ensuring safe use, optimal performance, and long‐term stability and durability; 2) Achieving moderate adhesion is also challenging. Although strong adhesion is necessary for stable electrode–tissue interfaces, excessive adhesion may damage tissue or cause discomfort during prolonged use or removal. Moderate bioadhesion provides sufficient stability for reliable signal acquisition and transmission and minimizes the risk of tissue injury. Such a balance is vital for neural interfacing applications, in which delicate neural tissues require careful handling to prevent damage. Therefore, understanding the interactions between hydrogel‐based HMI materials and biological tissues is crucial to optimize the adhesive properties and ensure stability in dynamic physiological environments. Key trends in mussel‐inspired and polyphenol‐mediated adhesion include suppressing catechol group oxidation during curing and adhesion processes through dynamic chemical reactions or structural design and developing responsive or adaptive hydrogels that can modulate their adhesive properties in response to environmental changes. These approaches also have potential for realizing moderate adhesion and ensuring long‐term stability with hydrogel‐based HMIs; 3) The complexity of fabrication is another significant issue. Normal synthesis techniques for hydrogels, including physical and chemical processes, have limitations in precisely controlling their adhesive, mechanical, biocompatibility, and conductive properties.^[^ ^146^ ^]^ Recently, advanced manufacturing technologies for hydrogel HMI electrodes include electrospinning,^[^ ^147^ ^]^ 3D printing,^[^ ^148^, ^149^ ^]^ laser patterning,^[^ ^150^ ^]^ layer‐by‐layer assembly,^[^ ^125^, ^151^ ^]^ and soft lithography.^[^ ^60^, ^144^ ^]^ However, each of these technologies has distinct advantages and limitations. For example, electrospinning is highly sensitive to various parameters such as voltage, feed rate, and solution viscosity, making it challenging to achieve consistent results. 3D printing allows the precise fabrication of complex electrode designs tailored for specific applications, offering high customization and scalability. However, the monomer materials for hydrogels must possess an appropriate viscosity for effective printing, which can limit the range of hydrogels that can be used. Laser patterning provides high‐resolution electrode structures, enabling fine control over the conductivity and surface properties of the hydrogels. Its advantages include precision and speed; however, the process may cause localized thermal damage to the hydrogel electrodes. Soft lithography involves creating patterned hydrogel surfaces using molds, offering a cost‐effective method for replicating intricate designs; however, long‐term stability remains a challenge. This layer‐by‐layer assembly process enables the assembly of different layers with distinct functions to create multifunctional HMIs. However, challenges remain in achieving uniform layer thickness and composition, which can lead to inconsistencies in the final properties of the hydrogel electrode. The connections between the different layers may not be sufficiently robust, potentially resulting in delamination or failure during use. Although these methods address some fabrication issues, they are still intricate and resource intensive, thereby limiting their scalability and increasing production costs. Ensuring uniform and reproducible performance across different batches of hydrogel electrodes is also difficult because variations in the synthesis process can lead to inconsistencies in the material properties, which affect the reliability of the corresponding bioelectronic devices; 4) The electrical properties, such as conductivity and impedance, must be tuned carefully to ensure effective signal transduction by the hydrogel electrodes without introducing significant noise or signal loss. In neural interfacing, distinguishing desired neural signals from background noise, particularly in the presence of electrically active tissues, remains a technical challenge, as does integrating these hydrogels with existing electronic systems and ensuring their compatibility with other device components. Customizing hydrogel electrodes by varying the stiffness, adhesion, conductivity, or self‐powering capabilities to meet the specific requirements of different bioelectronic applications can also be complex; 5) Establishing efficient wireless communication between implanted hydrogel electrodes and external devices through Bluetooth or NFC technology without using wires and providing a reliable, biocompatible power supply is another challenge. For active implantable devices, external wireless charging or self‐powered electrodes should also be considered; 6) Multifunctional hydrogels that integrate sensing, actuation, and data processing capabilities within a single platform are gaining increasing attention. This convergence of functionalities could lead to the development of sophisticated and efficient bioelectronic systems. Furthermore, emerging personalized medical approaches, in which hydrogel electrodes are tailored to the needs of individual patient, have the potential to improve treatment effectiveness and enhance patient outcomes; 7) Polyphenol‐mediated and mussel‐inspired multifunctional hydrogel‐based HMI electrodes have potential applications in regenerative medicine. These electrodes, which can support cell growth and regeneration, may even accelerate regeneration efficiency through the electrostimulation of integrated growth factors. This approach could aid in clarifying the effects of exogenous physical fields on cells, tissues, and organs, toward the development of new strategies for disease treatment based on physical stimulation.

Integrating multiple functionalities, such as sensing, actuation, and data processing, into a single hydrogel‐based system while maintaining the overall system performance is a complex engineering task. Addressing these challenges requires ongoing research and development to optimize the material properties, fabrication processes, and integration techniques toward realizing the full potential of polyphenol‐mediated and mussel‐inspired multifunctional HMI hydrogel electrodes in bioelectronics.

## 12 Conclusion

In this review, we have summarized recent advances in polyphenol‐mediated multifunctional hydrogel‐based HMI electrodes for bioelectronics. Key topics included the mechanisms of adhesion inspired by mussels, the mechanical properties that match biological tissues, and the electrodes’ electronic performance, biocompatibility, stability, and resistance to biofouling. Hydrogels, with their soft, flexible nature and high water content, are ideal for HMI electrodes, facilitating effective communication between machines and biological tissues. To ensure bidirectional communication between machines and biological tissues during bioelectronic applications, bioadhesion, conductivity, and tissue‐matching mechanical properties of hydrogel‐based HMI electrodes are particularly important.

Mussel‐inspired and polyphenol‐mediated adhesion mechanisms and strategies for endowing HMI electrodes with bioadhesion were outlined. Despite their benefits, challenges such as the oxidation of catechol groups and the need for moderate adhesion without causing damage must be addressed. The multifunctional properties of polyphenol‐mediated adhesive hydrogel‐based HMI electrodes were reviewed. The roles of conductivity, energy storage, and energy harvesting properties in bidirectional communication were considered. The mechanical properties of hydrogel electrodes can be tailored to match various tissues, as different parts of the body require HMI electrodes with different mechanical properties. Biofouling resistance and biocompatibility are crucial for the stable and long‐term use of hydrogel‐based HMI electrodes, as well as user comfort. The anti‐inflammatory and antioxidant properties are promising for in vivo applications, particularly in medical therapies for wound healing.

Potential applications include neural interfacing, wearable health monitoring, medical diagnostics, and energy‐harvesting devices. However, challenges such as achieving long‐term adhesion and integrating multiple functions in a single hydrogel remain. Future research should focus on optimizing material properties and integration techniques to enhance the capabilities of hydrogel‐based HMI electrodes, paving the way for advancements in bioelectronics and biomedical technologies. With continued innovation, these materials have the potential to transform various fields in medicine.

## Conflict of Interest

The authors declare no conflict of interest.

## Author Contributions

**Lili Jiang**: Writing—original draft (lead); Writing—review & editing (lead). **Donglin Gan**: Writing—original draft (supporting). **Chuangyi Xu**: Writing—original draft (supporting). **Tingting Zhang**: Writing—original draft (supporting). **Mingyuan Gao**: Writing—original draft (supporting). **Chaoming Xie**: Writing—original draft (supporting). **Denghui Zhang**: Supervision (supporting). **Xiong Lu**: Supervision (lead); Writing—review & editing (supporting).
